# Large-scale mapping of the MCH network in ALS mice reveals the vulnerability of dopaminergic and GABAergic neurons in zona incerta

**DOI:** 10.1186/s40478-026-02231-z

**Published:** 2026-02-06

**Authors:** Jelena Scekic-Zahirovic, Stefano Antonucci, Diana Wiesner, Chiara Ebner, Hussein El Hajj, Oumayma Aousji, Kareen Halablab, Yiting Fan, Anneka Zelaya, Gizem Yartas, Karthik Baskar, E. Anastasia Çakmak, David Bayer, Hoon-Ki Sung, Luc Dupuis, Jeehye Park, Francesco Roselli

**Affiliations:** 1 German Center for Neurodegenerative Diseases-DZNE, Ulm, Germany; 2 Department of Neurology, Ulm University, Ulm, Germany; 3 Molecular and Translational Neuroscience, Master program, Ulm University, Ulm, Germany; 4 University of Strasbourg, INSERM, Strasbourg Translational neuroscience & Psychiatry STEP–CRBS, UMR-S 1329, 67000 Strasbourg, France; 5 Genetics and Genome Biology Program, The Hospital for Sick Children, Toronto, ON Canada; 6 Department of Molecular Genetics, University of Toronto, Toronto, ON Canada; 7 Translational Medicine Program, The Hospital for Sick Children, Toronto, ON Canada; 8 Department of Laboratory Medicine and Pathobiology, University of Toronto, Toronto, ON Canada

**Keywords:** Amyotrophic lateral sclerosis, Weight loss, Melanin-concentrating hormone neurons, Monosynaptic rabies tracing, Zona incerta, Dopaminergic neurons

## Abstract

Weight loss and hypermetabolism are early and prognostically significant features of amyotrophic lateral sclerosis (ALS) and are associated with hypothalamic atrophy and degeneration of melanin-concentrating hormone (MCH) neurons that regulate energy balance. To investigate whether MCH vulnerability arises from upstream network dysfunction, we performed whole-brain retrograde rabies tracing in *SOD1*^*G93A*^ mice. We identified an early, selective loss of monosynaptic inputs from the zona incerta (ZI), a dopaminergic (DA)/gamma-aminobutyric acid (GABA)ergic nucleus that preceded MCH neuron degeneration. Neurochemical profiling confirmed the DA/GABAergic identity of these ZI input neurons, and ZI/DAergic neurons later degenerated. ALS-related pathology emerged early in the ZI, paralleling pathology in the motor cortex, while anterograde mapping revealed that motor cortical projections preferentially targeted the ZI, linking vulnerable motor and metabolic networks. Loss of ZI/DAergic neurons was observed in conjunction with weight loss in non-SOD1 ALS models. These findings identify the ZI as an early-affected node within hypothalamic networks and suggest that disruption of DA/GABAergic inputs to MCH neurons is associated with subsequent MCH and DA neuronal vulnerability, degeneration and metabolic imbalance in ALS.

**Supplementary Information:**

The online version contains supplementary material available at 10.1186/s40478-026-02231-z.

## Introduction

Amyotrophic lateral sclerosis (ALS) is primarily characterized by the degeneration of upper motor neurons (UMN) in the motor cortex and lower motor neurons (LMN) in brainstem and spinal cord, leading to progressive paralysis with unfavourable outcome [52]. While ALS is traditionally considered as an isolated motor neuron disorder, a growing body of evidence highlights systemic metabolic dysfunction as a clinically relevant disease feature, often preceding motor symptoms. ALS patients commonly present non-motor symptoms such as weight loss [29, 63, 81], hypermetabolism [9], and lipid disturbances [21, 50], which are independently recognized as negative prognostic factors for the disease [9, 36, 55, 72]. Several animal ALS models, in particular SOD1 transgenic mice, replicate metabolic phenotype prior to the development of motor deficit, further underscoring the link between metabolic alteration and the neurodegenerative process [51].

Metabolic dysfunction in ALS has been linked to hypothalamic pathology, as evidenced by the correlation between lower body mass index (BMI) and hypothalamic atrophy in ALS patients and in asymptomatic gene-carriers [28, 48, 53]. Furthermore, reduced hypothalamic volume has been associated with earlier disease onset and worse prognosis [14, 28]. The hypothalamus plays a central role in energy balance through a complex network of diverse neuropeptidergic neurons [1]. In line with the hypothalamic atrophy, post-mortem studies in ALS have revealed TDP-43 pathology in the lateral hypothalamic area (LHA) [19] as well as the degeneration of several subpopulations, including oxytocin, hypocretin/orexin, and melanin-concentrating hormone (MCH) neurons [7, 25]. However, selective pharmacological manipulation of hypothalamic subpopulations has yielded limited therapeutic benefits [7, 22, 31], although nutritional interventions have demonstrated the capacity to extend survival in ALS patients [49]. These findings collectively suggest a more extensive vulnerability of the hypothalamus in ALS, which remains to be thoroughly characterized in order to develop causal therapeutic strategies for metabolic dysfunction. In this context, initial evidence suggests that the architecture of large-scale networks that provide inputs to the metabolism-regulating LHA is affected in ALS patients and mouse models [5]. Nevertheless, which hypothalamic subpopulations are the direct projection targets within the affected network remains unknown.

We hypothesised that the ALS-related hypothalamus vulnerability may reside within a specific subnetwork rather than across individual neuropeptidergic subpopulation and that altered connectivity may contribute to the downstream degeneration and to the metabolic phenotype of ALS. In this study, we aimed to 1) determine the full scope of ALS-susceptible hypothalamic subpopulations, 2) selectively map the upstream networks of the most vulnerable subpopulation using genetic tools and viral tracers, and 3) characterise the earliest connectivity changes and their temporal progression across the disease stages along with mechanisms in ALS mouse models displaying distinct metabolic phenotypes.

## Materials and methods

### Mouse breeding and housing

All mouse experiments were performed in accordance with the relevant ethical regulations under The German Animal Welfare Act for animal testing and researchers and approved by the ethical committee of the Regierungspräsidium Tübingen (under the animal licence number #1522).

For retrograde tracing experiments, the double transgenic mice were generated by cross-breeding the B6SJL-Tg(SOD1*G93A)1Gur/J mouse line [32] from the Jackson Laboratory (#002726) and *Pmch-Cre* BAC transgenic mice [42] on C57BL/6 background (#014099), kindly provided by Prof. Dr Denis Burdakov. Heterozygous male mice *SOD1*^*G93A*^*;Pmch-Cre* and their healthy littermates *WT;Pmch-Cre* (F1 generation) received intrahypothalamic injections.

Genotyping of *Pmch-Cre* transgenic mice was performed by PCR using a 3-primer strategy: *Pmch-Cre* forward 5’GAA AAG ATA AGG CCT TCA AGT GCT, *Pmch-Cre* reverse 5’GAT CTT TCT GCA GTA TCT TCC TTC, and Cre-FR 5’ATC GAC CGG TAA TGC AGG CAA. These primers will generate two bands, a 300 bp band for the endogenous *Pmch* gene and a 200 bp band for the transgene, as previously described [42].

For histology *SOD1*^*G93A*^ and control *WT* mice were sacrificed at postnatal day P45 and at P110, according to the previously determined onset of motor and non-motor (weight loss) phenotypes [60]. The colony of *Fus*^*∆NLS/*+^ mice and their *WT* littermates were sacrificed at 12-months of age after the onset of motor symptoms [68].

Mice were group housed and routinely maintained in the animal facility under constant conditions (21 °C temperature; 60% humidity) and under a 12-h light/dark cycle (lights on at 7:00 am) with ad libitum access to standard laboratory mouse chow and water.

### Tissue preparation

Male mice were anaesthetised with an intraperitoneal injection of 100 mg/kg ketamine chlorhydrate (Ketamine 10%, WDT, Garbsen) and 5 mg/kg xylazine (Rompun 2%®, Bayer), and then transcardially perfused with 50 ml of ice-cold solution of 5 mM EDTA (PanReac AppliChem #A4892) in 1 × phosphate buffered saline (PBS, Gibco^TM^ #14,200,075), followed by 50 ml of 4% PFA (Sigma-Aldrich #441,244) solution in 1 × PBS for the fixation. After dissection, brains were post-fixed in PFA (4%) for 24 h, rinsed in 1 × PBS, and cryoprotected in 30% sucrose until dehydrated. Brains were then cryo-embedded in optimal cutting temperature compound (Tissue-Tek® O.C.T. Compound), frozen and stored at −80 °C until being sectioned (40 μm thickness for injected brains and 20 μm for ISH) on a cryotostat (Leica CM3050 S). Sections were stored in a cryoprotectant solution containing 30% ethylene glycol (ROTH #688.1,) and 20% glycerol (Fisher Chemical #EC 200-289-5) at −20 °C.

### Preparation of brain tissue from ***Matr3***^***S85C/S85C***^ knock-in mice

Brain samples were prepared in Dr. Jeehye Park lab and then shipped to Dr. Francesco Roselli lab for further processing. Homozygous *Matr3*^*S85C/S85C*^ and wild-type littermate *Matr3*^+*/*+^ mice [39] at 5060 weeks of age were deeply anaesthetized using gas isoflurane and then transcardially perfused in a same way as written above. After dissection and post-fixation brains were shipped in 1 × PBS containing 0.05% of sodium azide (Sigma-Aldrich #S2002).

### RNAscope assay – single-molecule mRNA fluorescence multiplex in situ hybridisation

The single-molecule mRNA fluorescence multiplex in situ hybridization was performed in accordance with the manufacturer’s UM instructions (Advanced Cell Diagnostics–ACD, Newark, CA) and as previously reported [77]. The selected brain coronal sections, containing the hypothalamus region, were washed in RNase-free 1 × PBS, mounted on Superfrost Plus slides and after air-drying were stored overnight at −80 °C.

For the assessment of the neuropeptidergic hypothalamic population, the Sects. (20 μm thick) of *WT* and *SOD1*^*G93A*^ colony were processed using the RNAscope® Fluorescent Multiplex Reagent Kit (containing all reagents/buffers, ACD, Newark, CA) and the method reported by the manufacturer (protocol 320,293-USM for fixed frozen tissue sections, ACD) with minor adjustments. Briefly, following air-drying and washing in RNase-free 1 × PBS for 5 min, the target retrieval was performed by immersion in the boiling target retrieval solution (95–98 °C) for 5 min and the reaction was stopped by dipping twice in RNase-free H_2_O and once in 100% ethanol. The protease digestion step was then carried out inside a preheated HybEZ oven at 40 °C using RNAscope Protease III for 20 min and the reaction was stopped by dipping twice in RNase-free H_2_O. The hybridization using the corresponding target RNA probe was performed at 40 °C for 4 h 30 min (the catalogue numbers of the used RNA probes are provided in the Supplementary Table 1). Subsequently, the slides were washed twice with washing buffer for 5 min and the amplification steps were performed by incubation with amplification-1 reagent at 40 °C for 30 min, then amplification-2 reagent at 40 °C for 15 min and finally with amplification-3 reagent for 30 min at 40 °C. Between each step the slides were washed twice for 5 min each. The final amplification—detection step was performed by incubation in amplification-4 reagent at 40 °C for 45 min, followed by two washing steps, each extended to 10 min.

In order to assess the neurochemical identity of neurons projecting to MCH, the Sects. (40 μm thick) of *WT;Pmch-Cre* and *SOD1*^*G93A*^*;Pmch-Cre* colony were processed using RNAscope Multiplex Fluorescent Reagent Kit v2 in conjunction with TSA Vivid Dyes (ACD, Newark, CA) and in accordance to the manufacturer's protocol (UM 323100/Rev B, ACD), with slight optimisations. In summary, following air-drying and the two washes in RNase-free 1 × PBS for 5 min each, the sections were preheated in a HybEZ oven at 60 °C for 30 min. Thereafter, the sections were post-fixed with pre-chilled 4% PFA in RNase-free PBS for 15 min at 4 °C. Subsequently, the slides were dehydrated using the increasing ethanol solutions in RNase-free 1 × PBS of 50%, 70% and twice of 100% during 5 min each at room temperature. The sections were then treated with RNAscope Hydrogen Peroxide for 10 min at room temperature (RT) and rinsed three times in RNase-free H_2_O. The target retrieval and the protease digestion steps were then carried out as described above. The target probe for tdTomato-C2 was mixed with either of the target probes for VGAT-C3 (*Slc32a1*), VGlut2-C3 (*Slc17a6*) or Th-C3 (tyrosine hydroxylase) and diluted in Probe diluent to 1:100 each. The hybridization step was shortened to 2 h, while the amplifications steps 1, 2 and 3 remained the same as noted above. The final detection step was modified and repeated for each of the used probes separately. The sections were first incubated in the oven with HRP-C3 for 15 min at 40 °C, followed by two washes for 5 min each. Subsequently, a TSA Vivid Dye-650 diluted in TSA buffer (1:3000) was added to the sections, which were then incubated for 30 min at 40 °C. Following two washes of 5 min, an HRP blocker was added to the sections and they were incubated for 15 min at 40 °C, followed by two washes for 5 min each. The procedure was repeated using HRP-C2, TSA Vivid Dye-520 (1:3000) and HRP blocker and the final washing step was extended to 10 min.

At the end of the procedure, the slides were mounted using ProLong Gold Antifade Mountant (Invitrogen #P36930) and a glass coverslip was placed over the tissue section.

### Viral vectors

The following helper viruses were used for cell type-specific retrograde tracing: AAV1 expressing pAAV-syn-FLEX-splitTVA-EGFP-tTA, at titer 1.3 × 10^13^ vg/ml (viral genomes per ml) (Addgene #100,798; Watertown, MA) [47] and AAV2 expressing pAAV-CAG-DIO-oG-WPRE, at a titer of 1 × 10^12^–1 × 10^13^ vg/ml (the virus was produced by Vector Biosystems, Malvern-PA, US; using Addgene #74,292) [40], the plasmid was kindly donated by Edward M. Callaway. A pseudotyped rabies virus expressing mCherry in the place of the rabies glycoprotein N2C RVΔG/EnvA-mCherry [2], at a titer of 2 × 10^8^ vg/ml was a kind gift from Prof. Dr. Karl-Klaus Conzelmann.

For the anterograde tracing, the following custom-made AAV2/9 viruses were used encoding pAAV-AiP1010-mscRE4-minBGpromoter-iCre-WPRE-hGHpA, at a titer of 1.4 × 10^13^ vg/ml, (Addgene #163,476) [30], and pAAV-phSyn1(S)-FLEX-tdTomato-T2A-SypEGFP-WPRE, at a titer of 5 × 10^12^ vg/mL (Addgene #51,509) [58].

### AAV production and titration

AAVs were produced in-house using the triple transfection method as previously described [38]. HEK293T cells were maintained in DMEM supplemented with 10% fetal bovine serum (FBS) and 1% penicillin–streptomycin-glutamine at 37 °C in a humidified incubator with 5% CO_2_. For virus production, cells were expanded to thirty 15 cm dishes and transfected at 70–80% confluence with polyethyleneimine (PEI; Polysciences #23,966) in a 1:1:1 ratio of plasmids: an AAV2/9 capsid plasmid [75] (kindly provided by Prof. Dr. Oliver J. Müller), a vector genome plasmid (either Addgene #163,476 or #51,509), and the helper plasmid pAdDeltaF6 (Addgene #112,867). The plasmid-PEI complexes were prepared in 0.3 M NaCl, incubated for 15 min at room temperature, and added dropwise to the culture dishes. Cells were harvested 72 h post-transfection, pelleted at 3000 × g for 5 min at 4 °C, and lysed in lysis buffer (1 × EDTA, 1 × protease inhibitor, pH 8.5). Supernatants were filtered through 0.22 µm PES filters, and viral particles were precipitated with ammonium sulphate (330 g/L) for 2 h at 4 °C. The resulting pellet was combined with the cell lysate, subjected to four freeze–thaw cycles (liquid nitrogen/37 °C water bath), and treated with benzonase nuclease (50 U/mL, 45 min, 37 °C; Sigma-Aldrich #E1014-25KU,). Lysates were cleared by centrifugation (3000 × g, 5 min, 4 °C), and the supernatant was stored at 4 °C.

Virus purification was performed using iodixanol step gradient ultracentrifugation [41]. Gradients (15%, 25%, 40%, and 60%) were prepared in Ultra-Clear^TM^ tubes and centrifuged at 200,000 × g for 2 h at 18 °C using a Beckman Ti 70 rotor. The 40% iodixanol fraction, containing the AAV particles, was collected by syringe, avoiding the protein-rich and empty capsid layers. For buffer exchange, the viral fractions were passed through Zeba^TM^ spin desalting columns (Thermo Fisher #A57766) pre-washed with DPBS + / + . Concentration was performed using Vivaspin® 100 kDa PES filters (Sartorius), pre-treated with 0.01% Pluronic-F68 in DPBS + / + and equilibrated with formulation buffer (200 mM NaCl, 0.001% Pluronic-F68 in DPBS + / +). Final virus suspensions were aliquoted and stored at −80 °C. Flow-throughs were retained as negative controls for titration. Viral genome titers were determined by qPCR.

### Surgery and stereotaxic injection—Intrahypothalamic injection of AAV and modified rabies virus for retrograde tracing

Intrahypothalamic injection of AAVs or modified rabies virus was performed as previously described with slight modifications [5]. Mice were pre-treated with a combination of the analgesic buprenorphine (0.1 mg/kg) and the anaesthetic meloxicam (1 mg/kg) 5 min before induction of anaesthesia with 5% sevoflurane/95% O_2_. Mice were mounted in a stereotaxic frame (David Kopf Instruments, Tujunga, CA) and ophthalmic ointment was applied. Throughout the procedure, anaesthesia was maintained continuously with 2% sevoflurane/97% O_2_, and body temperature was maintained at 37 °C with a heated pad and monitored with a rectal probe. After scalp incision, craniotomy was performed manually with a microdrill and 700 nl of a mixture containing viral suspension of helper viruses: AAV1-syn-FLEX-splitTVA-EGFP-tTA (diluted 1:10 in + Ca/ + Mg Dulbecco's PBS (Thermo Scientific^TM^ #14,080,048)) and AAV2-CAG-DIO-oG-WPRE (diluted 1:2) in 1% Fast Green dye, prepared in a 1:1:2 ratio, was injected with a pulled-glass capillary micropipette at the LHA coordinates (1.30 mm caudal to bregma; 0.85–0.9 mm lateral to midline; 5.3 mm, 5.0 mm and 4.7 mm ventral to the pial surface of the brain). The viral suspension was injected using a Picosprizer microfluidic pressure injection pump for 18 min at a pressure of 40 psi, a flow rate of 40 nl/min and a pulse duration of 10 ms. After injection, the capillary remained in place for 10 min preventing backflow, prior to retraction, after which the incision was closed with 7/0 nylon suture thread (Ethilon Nylon Suture, Ethicon Inc. Germany), and the mice were kept warm on a heating pad until complete recovery. After 30 days, a second injection this time of N2C RV-ΔG-EnvA-mCherry in 1% Fast Green dye, prepared at a 1:1 ratio, was injected into the same LHA sector over 12 min (500 nl), and the mice were sacrificed 8 days later. All injections were placed in the right hemisphere. After each intrahypothalamic injection, mice were administered with a daily dose of 1 mg/kg meloxicam and three daily doses of 0.1 mg/kg buprenorphine for the next 3 days. Two cohorts of early-symptomatic mice (first injection at P50) were injected with different doses of modified rabies virus: LT-low titer (RVΔG/EnvA-mCherry, diluted 1:10, 1 × 10^7^ vg/ml) and HT-high titer (undiluted, 1 × 10^8^ vg/ml), while late-symptomatic mice (first injection at P75) received only the low dose of modified rabies.

### Intracortical AAV injection for anterograde tracing

Intracortical injection of the AAV suspension was performed in mice aged P70 in correspondence with the stereotaxic coordinates of the motor cortex (0.7 mm caudal to bregma; 0.7 mm lateral to midline; 1.0 mm and 0.7 mm ventral to the pial surface of the brain), as previously described [17]. An amount of 300 nl of a mixture containing viral suspension of AAV2/9-AiP1010-mscRE4-minBGpromoter-iCre-WPRE-hGHpA and AAV2/9-phSyn1(S)-FLEX-tdTomato-T2A-SypEGFP-WPRE in 3% Fast Green dye, prepared at a 2:2:1 ratio, was injected into motor cortex over 10 min. Pre- and post-treatment of the mice was carried out as described above. The mice were sacrificed 30 days later.

### Whole-brain connectome analysis

The fixed brains were serially sectioned at 40 μm thickness and every second coronal brain Sect. (90 per animal) was mounted on the glass slide. Each section was individually scanned using an epifluorescence microscope (Keyence, BZ-X800E series); to create a seamless whole-brain dataset, a custom *Python* script was run to stitch individual tiles with the Grid/Collection stitching plugin [64] and perform shading correction with the BaSic plugin [62] in *Fiji* [69]. Brain section stencils were outlined upon Li thresholding [44] and pasted into black canvas images of defined dimensions (16,000 × 11,000 px) to account for minor discrepancies arising from stitching calculations and to facilitate downstream analysis. Finally, brain sections were registered to the CCFv3 Allen Brain Mouse reference atlas using *WholeBrain* R package [24] (http://wholebrainsoftware.org). First, we determined the coordinates along the anterior–posterior axis using the Openbrainmap website [24] (http://openbrainmap.org) for sections with recognizable anatomical landmarks; all sections in between were automatically mapped in such a way that they were equidistant from each other and the closest landmarks, ensuring spatial consistency. Next, following the standard *WholeBrain* workflow, anatomical landmarks were manually annotated to all sections to enable spline-based registration of the atlas plate of choice onto the brain section, generating a warping field; visual inspection of the superimposed plate outlines confirmed satisfactory anatomical annotation. Since the sectioning plane or disease-driven atrophy or select brain areas could make sections deviate from the reference plates, hemispheres and individual regions were annotated in different rounds of registration. Initially, neurons were segmented using the inbuilt tunable settings of the *WholeBrain* software (feature size, sphericity and signal intensity). However, given the limited accuracy of the detection filters, only neurons that were accurately mapped to the regions under analysis were retained. False positives (artefacts) were recognized upon visual inspection of segmentation results and removed from results tables, whereas the image coordinates of the false negatives were retrieved in *Fiji* and, after matching *Fiji* and *WholeBrain* image coordinate systems, added to the results tables with the correct anatomical annotation. The process was then repeated until exhaustion of the MCH brain-wide inputs. Finally, edited results tables were imported back in R to generate accurate 3D reconstructions of brain-wide connectivity with *WholeBrain* in-built functions.

### Immunofluorescence free-floating immunolabelling on mouse brain sections

Immunofluorescence staining was performed on free-floating brain coronal Sects. (40 μm thick) of *WT* and *SOD1*^*G93A*^ samples as previously described [46]. Briefly, sections were washed in RNAse-free 1 × PBS, 3 × 30 min and subsequently incubated in blocking solution (3% BSA, 0.3% Triton-100 X, 1 × PBS or 3% Donkey serum (Sigma-Aldrich #D9663), 0.3% Triton-100X, 1 × PBS) for 2 h at RT. Afterwards the sections were incubated with primary antibodies (Supplementary Table 2) diluted in blocking solution O/N at 4 °C. The following day the sections were washed 3 × 30 min followed by incubation with secondary antibodies (Supplementary Table 2) diluted in blocking solution for 2 h at RT. After the final washing (3 × 30 min) the sections were mounted with a Prolong antifade mounting medium.

### Epifluorescence and confocal image acquisition and image analysis

Images were acquired using an epifluorescence microscope (Keyence BZ-X800E) with a 20 × (NA 0.75) objective in an 8-bit format of 1024 × 1024-pixel, except noted otherwise. A tile scan of images to cover the whole hypothalamus with 1.0 optical zoom and the same number of stacks (optical sections) per genotype with a z-step size of 1 µm, was stitched and collapsed into maximal intensity projection (MIP) images by the Bioanalyzer software (BZ-X800 analyser). Image analysis was performed using *Fiji* [69].

Neuropeptidergic populations were counted manually after the *in-situ* hybridization, using overview images of the entire hypothalamus and only neurons with DAPI nuclear staining in the same plane were included. For each animal neuropeptidergic populations were counted on the three-brain slice, matched between genotypes and separated by 100 µm in the rostro-caudal direction. The cumulative number of three slices was considered for analysis.

To assess the neurochemical identity of ZI inputs to the MCH, overview images of the ipsilateral hypothalamus (the injection side) were taken first, followed by a single neuron acquisition with a 100 × (NA 1.45) oil immersion objective and 25 stacks with a z-step size of 0,5 µm. The neurochemical identity was identified as the co-expression of *tdTomato* mRNA with *Slc32a1* mRNA, *Slc17a6* mRNA, and *Th* mRNA, respectively within the same neuron.

The DDC + and TH + neurons were manually counted in the ZI area on both sides and only neurons with DAPI nuclear staining in the same plane were considered. For each animal neurons were counted on the six-brain sections matched between genotypes and separated by 80 µm in rostro-caudal direction. The cumulative number of six sections was considered for analysis. The same was done for TH + neurons’ quantification in *Fus*^*∆NLS/*+^ mice on three brain slices separated by 120 µm.

The *Th* + neurons were quantified after the in-situ hybridization, using overview images of the entire hypothalamus and on three brain slices separated by 120 µm. The same was done for *Th* + neurons’ quantification in *Matr3*^*S85C/S85C*^ mice.

To quantify the B8H10 (hSOD1) + area, one overview image of the entire hypothalamus per animal was analyzed. For each image, the background was subtracted by the rolling-ball method (50 pixels diameter) in the green channel and the B8H10 (hSOD1) + area was quantified on the same image in three structures: thalamus (TH), zona incerta (ZI) on both sides and hypothalamus (HY) on both sides in the selected regions of interest (ROI). The rectangular area of the ROI was adjusted according to the area of the ZI and then the same ROI was used for TH and HY. The ROI images were thresholded to the same values for all images (57-255 for P45, and 46–255 for P110) and the total surface area above the threshold was computed. The absolute values of the area occupied by the B8H10 (hSOD1) + signal were normalized to the mean value of thalamus and reported as a percentage.

For the quantification of P62 + area, one overview image of the entire hypothalamus per animal was analyzed. For each image the background was subtracted by the rolling-ball method (100 pixels diameter) in the corresponding channel and the P62 + area was quantified on the same image in ZI and LHA, on one side of the hypothalamus in the selected ROI. The rectangular area of the ROI was adjusted according to the area of the ZI or to the area of the LHA, then the same ROI was used for *WT* and *SOD1*^*G93A*^ animals. The ROI images were thresholded to the same values for all images (55-255 for ZI, and 50-255 for LHA) and the total surface area above the threshold was calculated. The absolute values of the area occupied with P62 + signal was normalized to the average value of *WT* animals for the ZI and LHA and expressed as a percentage.

For the quantification of GFAP + and IBA1 + area, two overview images of the entire hypothalamus per animal were analysed for P45, and three images were analysed per animal for P110. Images were analysed without the background subtraction. The area of the ROI was adjusted according to the whole hypothalamus (HY) or to the area of the ZI, then the same ROIs was used for *WT* and *SOD1*^*G93A*^ animals. The ROI images were thresholded to the same values for all images (121-255 for GFAP, and 135-255 for IBA1) and the total surface area above the threshold was computed. The absolute values of the area occupied by GFAP + or IBA1 + signals were first calculated as a percentage of the corresponding ROIs’ area of HY or ZI and then normalized to the average value of *WT* animals for the HY and ZI and reported as a percentage. The same was done for GFAP + area quantification in *Fus*^*∆NLS/*+^ mice.

To assess the starter cells each hypothalamic hemi-section, separated by 80 µm (used in the tracing study and with natively expressed reporter proteins–no staining) was individually scanned, tiles were then stitched together and resized using a custom macro in *Fiji* [69]. GFP + neurons (expressing TVA) and double positive GFP + /mCherry + (starter cells) were counted manually on those sections where both reporters were visible (approximately 20–25 sections per animal).

Confocal imaging was performed using a laser scanning confocal microscope (Leica DMi8) in order to assess synaptic contacts between cortical L5PN inputs to MCH and to TH neurons as well as to evaluate synaptic contacts between TH and MCH neurons. In confocal images, the laser power was set in the range of 5–15%. images were acquired with a 63 × (NA 1.3) oil immersion objective in 12-bit format 1024 × 1024 pixels, as a z-stack of 40 optical sections spanning 14 μm (step size of 0.35 μm) and with an optical zoom of 1.5. The area occupied by GFP + puncta was quantified on 8-bit format MIP images (4–5 images per animal), after thresholding to the same values for all images (80-255 for GFP). The total surface area above the threshold was computed and the absolute values of the area occupied by GFP + signal were normalized to the mean value of *WT* animals and represented as a percentage. The synaptic contacts, colocalization were inspected visually using orthogonal view images.

### Statistics

GraphPad Prism 9 (GraphPad, CA), and RStudio (version 4.2.2, (http://www.R-project.org)) were used to perform data analyses. Statistical analysis was performed using animals as a biological unit; whenever appropriate, single datapoints and averages per animal are depicted in the graphs. Statistical tests and descriptive statistics were performed as specified in the figure legends and in the text. An unpaired t-test was used for comparing two groups and grouped analysis was performed using 2-way ANOVA followed by Bonfferoni’s multiple comparisons test. The data were shown as the mean ± standard error of the mean (SEM) and p values smaller than 0.05 were considered significant.

## Results

### Loss of hypothalamic MCH, CART, POMC and galanin neurons occurs upon weight loss in late-symptomatic ***SOD1***^***G93A***^ mice

Since spatially-distinct neuronal subpopulation with a similar neurochemical identity may display differential vulnerability to disease, we elected to determine the size of multiple peptidergic subpopulations in the hypothalamus of male *SOD1*^*G93A*^ ALS mice using multiplexed in-situ hybridization. We considered two time points in the disease progression [60]: pre-symptomatic mice (prior to weight loss and prior to muscle denervation; P45) and late-symptomatic mice (displaying on average 30% weight loss; P110). Two anatomical regions of the hypothalamus were considered for neuronal quantification: the lateral hypothalamic area (LHA) and the arcuate nucleus (ARH), both of which are known to be involved in energy homeostasis [1, 10]. We selected two sets of subpopulations: 1) neurons stimulating food intake and promoting weight gain (such as MCH [7], cocaine- and amphetamine-regulated transcript (CART) in the LHA [43], agouti-related peptide (AgRP) [76], neuropeptide Y (NpY), Galanin (GAL) [66] and somatostatin (SST) [71]); and 2) neurons suppressing appetite and increasing energy expenditure (such as orexin/hypocretin (HCRT) [27], CART in the ARH [43] and pro-opiomelanocortin (POMC) [76]), (for a review, see [13]).

**Fig. 1:**
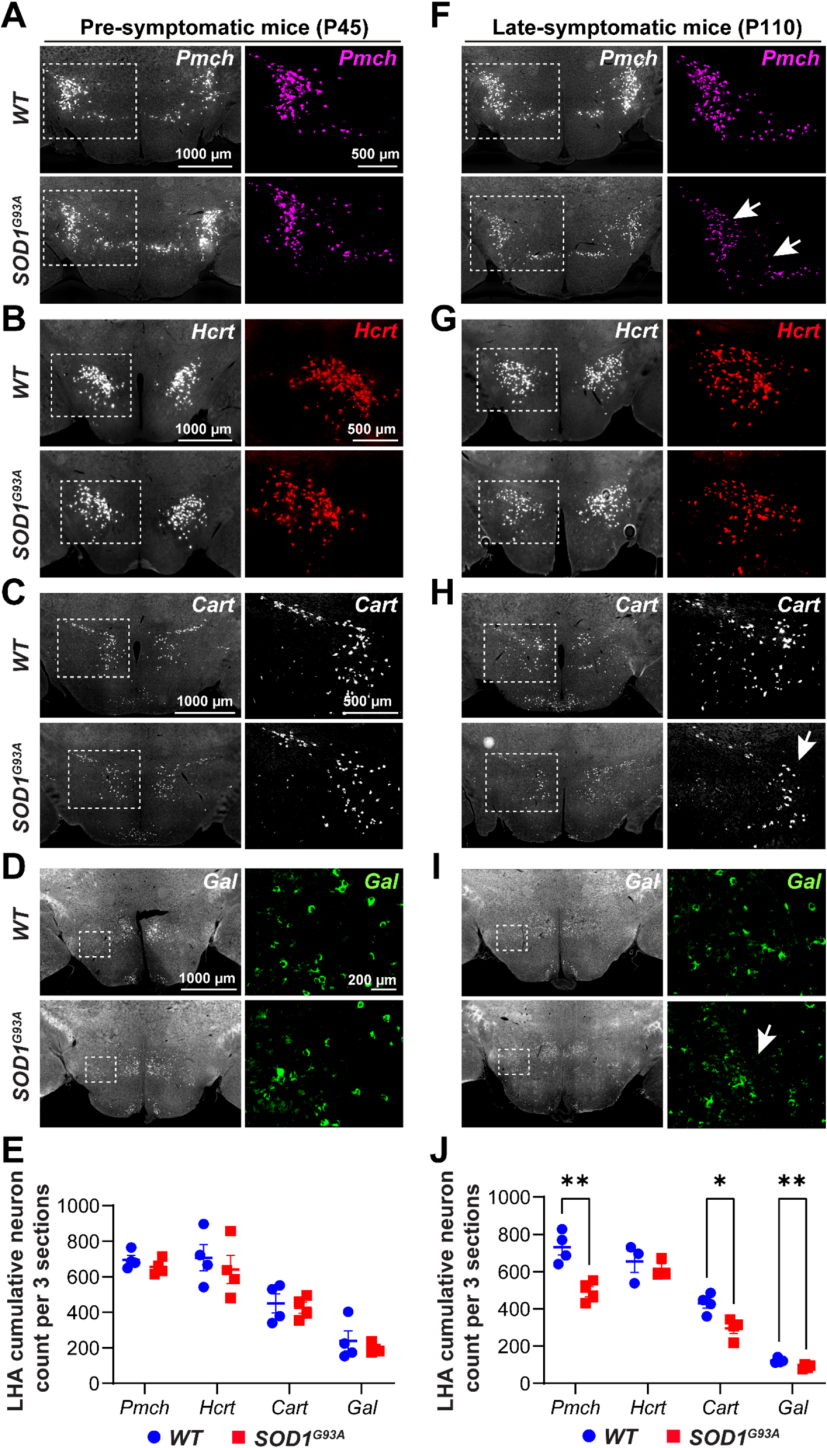
Prominent loss of hypothalamic MCH neurons in late-symptomatic *SOD1*^*G93A*^ mice. **A–D, F–I** Representative images showing the characteristic spatial distribution of the major hypothalamic neuropeptidergic subpopulations in the LHA, as detected by a RNAScope assay in *WT* (upper panels) and *SOD1*^*G93A*^ (lower panels) mice at 45 days of age (P45), prior to the weight loss **A–D**, and at 110 days of age (P110) after the weight loss **F–I**. For each neuropeptidergic subpopulation, the left panels present a coronal section of the hypothalamus (overview) while the right panels show enlarged insets (white dashed line rectangle) of the region of interest (neurons). The neurons express mRNA that encodes the corresponding neuropeptide: **A, F.** *Pmch* for melanin-concentrating hormone (MCH) (in magenta), **B, G.** *Hcrt* for orexin/hypocretin (in red), **C, H.** *Cart* for cocaine- and amphetamine-regulated transcript (in grey) and **D, I.** *Gal* for galanin (in green). **E, J** The graphs present the cumulative number of neurons counted on three sections in *WT* (blue) and *SOD1*^*G93A*^ (red) mice at P45 **E** and at P110 **J**. * Pmch* (*p* = 0.003), *Cart* (*p* = 0.01) and *Gal* (*p* = 0.009) expressing neurons are significantly reduced in *SOD1*^*G93A*^ mice at P110–late-symptomatic disease stage. N = 4 animals per genotype (except N = 3 animals per genotype for *Hcrt* at P110). (*) *p*-value < 0.05, (**) *p*-value < 0.01, Multiple unpaired t-test per neuronal population. Data are displayed as the mean ± standard error of the mean (SEM), with each data point representing a single animal. Scale bars: 1000 μm (for lower magnification images) and 200 μm (for higher magnification images)

At pre-symptomatic disease stage (P45), we detected no significant change in the number of any of the considered subpopulations in the *SOD1*^*G93A*^ mice compared to *WT* littermates (Fig. 1A–E; Fig. S2A–G). On the other hand, late-symptomatic *SOD1*^*G93A*^ mice (P110) displayed a significant decrease in *Pmch* + (approximately − 33%), *Cart* + (approximately − 31%) and *Gal* + neurons (approximately − 20%) in the LHA (Fig. 1F, H, I, J). Furthermore, the *SOD1*^*G93A*^ mice also showed a decline in *Pomc* + neurons (approximately − 10%) and in *Cart* + (approximately − 20%) in the ARH (Fig. S2J, K, N). All other subpopulations were preserved at P110 (Fig. 1G; Fig. S2H, I, L, M).

Thus, the spectrum of hypothalamic subpopulations vulnerable to degeneration in the *SOD1*-ALS mouse model is broader than previously reported and also includes CART, GAL and POMC neurons.

### Retrograde modified rabies tracing to selectively target monosynaptic inputs to MCH neurons

Since multiple neuronal subpopulations are vulnerable in the SOD1 hypothalamus, we hypothesised that the vulnerability may reside in a specific subnetwork rather than in any neurochemically-identified neuronal subpopulation. To address this hypothesis, we set out to define the network of neurons providing input to the MCH neurons (being these the most vulnerable).

To identify neurons that establish direct presynaptic connections to MCH neurons we employed monosynaptic retrograde tracing based on modified rabies virus [40, 82]. The starter population of MCH neurons was genetically targeted by an intersectional genetic strategy and crossing *SOD1*^*G93A*^ with *Pmch-Cre* BAC transgenic mice (Fig. S2A).

We delivered two helper adeno-associated virus (AAV) vectors (coding for the TVA and oG proteins under the DIO) in the LHA of mice either at P50, before the weight loss, (in order to investigate the early-symptomatic stage at P88) or at P75 (in order to investigate the late-symptomatic stage at P113, which coincides with MCH loss) (Fig. S2A). The RV∆G/ENV- mCherry, pseudo-typed and modified rabies virus, was then injected 30 days after the AAV, and the animals were sacrificed 8 days after the rabies injection (Fig. S2A). AAV-infected MCH + neurons expressed the GFP tag, whereas the rabies-infected neurons expressed the mCherry tag; therefore, starter neurons were GFP + /mCherry + while retrogradely labelled neurons (projections to MCH + neurons) were only mCherry + (Fig. S2B–D). As previously reported, only a fraction of neurons transduced with helper viruses was co-infected with rabies [83].

We confirmed the efficiency of the AAV/rabies co-infection in a late-symptomatic group of mice receiving AAV co-injection at P75. The AAV infected neurons (GFP +) and starter neurons (GFP + /mCherry +) were localized in the LHA, as predicted by the expression of MCH, and were comparable in number (150–300 per mouse, approx. 45% of the GFP +) in *WT* and *SOD1*^*G93A*^ mice (Fig. S3A–E). Furthermore, the presence of single-positive mCherry + neurons was confirmed either within the hypothalamus or in distant extra-hypothalamic regions confirming the short-range and long-range propagation of the rabies tag expression (Fig. S4A).

### Whole-brain and inputs from large-scale brain regions to MCH neurons are preserved in early-symptomatic ***SOD1***^***G93A***^ mice

We exploited the aforementioned rabies system to quantitatively map the inputs to MCH neurons at the two timepoints, before and simultaneously with the appearance of MCH loss. Retrogradely-labelled neurons were mapped to a large-scale brain reference atlas using the *WholeBrain* software [24] (see the Methods section).

**Fig. 2:**
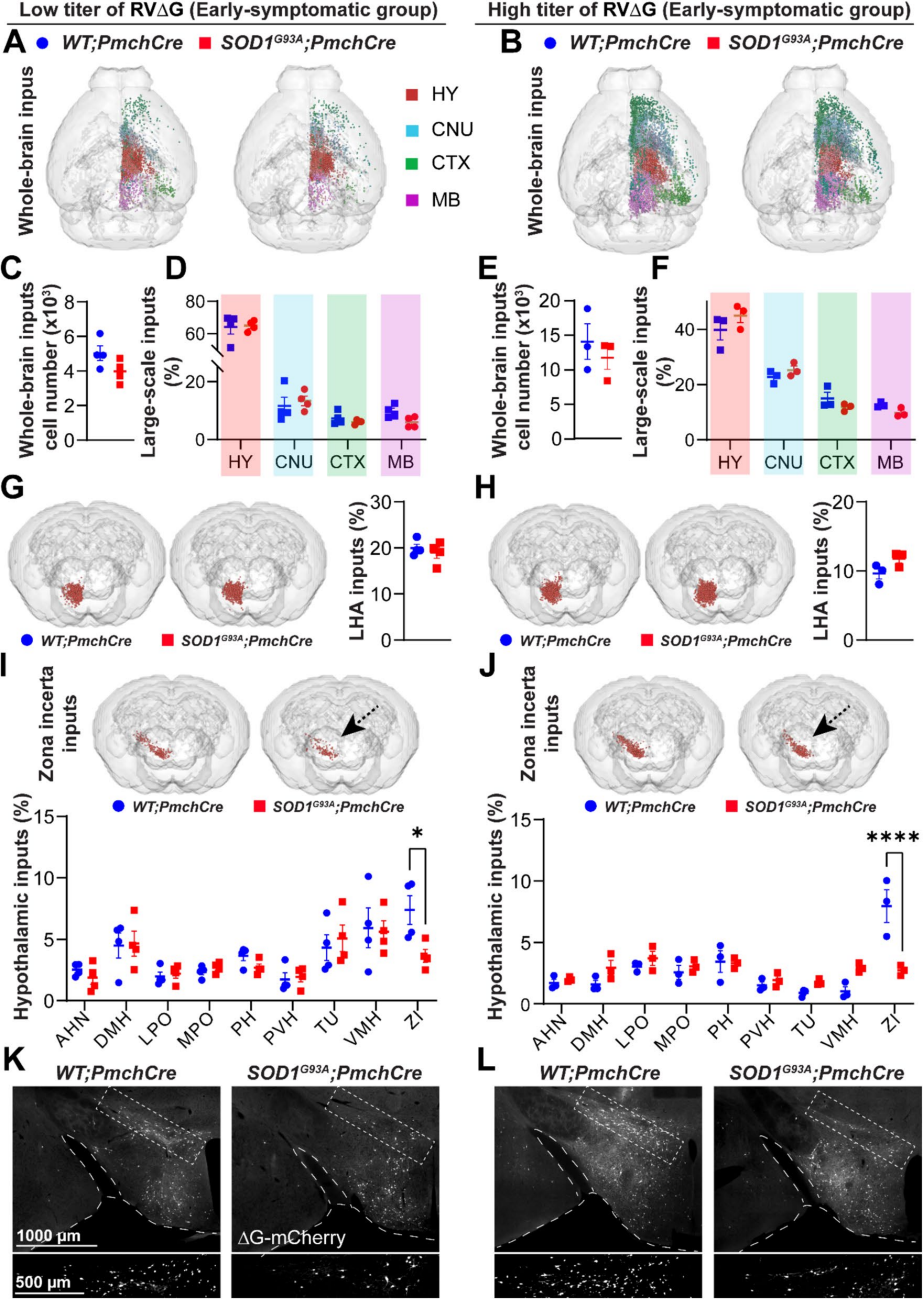
Whole-brain and HY inputs to MCH neurons in early-symptomatic *SOD1*^*G93A*^*;Pmch-Cre* mice. **A-B** Representative *WholeBrain* 3D reconstructions of neurons projecting to the MCH in *WT;Pmch-Cre* and *SOD1*^*G93A*^*;Pmch-Cre* mice upon receiving a LT **A** and HT **B** of rabies virus. Inputs are colour-coded according to their location within large-scale brain regions (red for HY, light blue for CNU, green for CTX and purple for MB). **C, E** Total sum of monosynaptic inputs (neurons) from all brain regions to the MCH in *WT;Pmch-Cre* (blue) and *SOD1*^*G93A*^*;Pmch-Cre* (red) injected with an LT **C** or HT **E** of rabies virus. No difference between genotypes was observed for either of viral titer. **D, F** The distribution of inputs across the large-scale brain regions. The difference between genotypes was not significant for any of the brain regions or viral titres, LT **D** or HT **F**. **G-H** 3D reconstructions and quantification of LHA inputs were comparable between genotypes. **I-J** 3D reconstructions of ZI inputs and the distribution of inputs across the selected meso-scale hypothalamic areas. A significant decrease in projections from the ZI was detected in *SOD1*^*G93A*^*;Pmch-Cre* mice (*p* = 0.02, for LT rabies **I**, and *p* < 0.0001, for HT rabies **J**, as indicated by the black dashed arrow on the 3D brains. **K-L** Representative images of the hypothalamus (at Bregma −1.3) showing the location of input neurons expressing the mCherry reporter (white) coupled with the rabies virus. The enlarged insets (white dashed line rectangle) depict ZI inputs, and a loss of mCherry + neurons in *SOD1*^*G93A*^*;Pmch-Cre* mice (left panels) in both LT **K** and HT **L** virus conditions. N = 4 animals per genotype for LT and N = 3 for HT virus concentration (*) *p*-value < 0.05, (****) *p*-value < 0.0001, Two-tailed unpaired Student’s t-test in **C, E, G** and **H** for comparison between two genotypes and 2-way ANOVA in **D, F, I** and **J** followed by multiple comparisons tests. Data are displayed as the mean ± SEM of neuron percentages found per region/area, except for absolute values **C** and **E**, with each data point representing a single animal. Anatomical abbreviations are based on the Allen Brain Atlas (ABA), and are fully defined in Supplementary Table 3. Scale bars: 1000 μm (for lower magnification images) and 500 μm (for higher magnification images)

Since retrograde efficiency is a function of the rabies viral load [37, 74] we mapped neurons projecting to MCH under two rabies load conditions: low titer (LT, 1 × 10^7^ vg/ml) and high titer (HT, 1 × 10^8^ vg/ml). We started our connectivity analysis at the early-symptomatic stage (P50-AAV inj.; P80-RV∆G inj.; P88-sacrifice, under the hypothesis that alterations of network architecture may appear before the actual degeneration of vulnerable MCH neurons. In LT and HT groups, the vast majority of input neurons resided on the hemisphere ipsilateral to the rabies injection, in agreement with previous reports [26] (Fig. 2A, B).

The number of labelled neurons in the entire brain hemisphere did not differ between genotypes, with the HT cohort exhibiting a greater number (Fig. 2C, E). A further examination of monosynaptic inputs was conducted, with allocations to four large-scale brain regions and their subregions. To facilitate comparison between cohorts and to account for inter-animal variability, the number of input neurons in each region was normalized. In both cohorts, the hypothalamus (HY) constituted the primary source of inputs to MCH neurons. A substantial proportion of inputs originated from the deep cerebral nuclei (CNU), with smaller contributions from the cortex (CTX) and midbrain (MB) (Fig. 2D, F). There were no evident differences in MCH neuron large-scale connectivity between *SOD1*^*G93A*^*;Pmch-Cre* and *WT;Pmch-Cre* mice at the early-symptomatic stage of disease.

It is noteworthy that a higher titre of rabies resulted in the redistribution of inputs to MCH neurons, leading to a reduction in the ratio of HY inputs (approximately 64% *WT* and 65% *SOD1*^*G93A*^ of all labelled neurons in LT and 40% *WT* and 45% *SOD1*^*G93A*^ in HT cohort), and to an increase in the proportion of CNU (12% *WT*; 13% *SOD1*^*G93A*^ in LT vs 23% *WT*; 25% *SOD1*^*G93A*^ in HT), CTX (7% *WT*; 6% *SOD1*^*G93A*^ in LT vs 15% *WT*; 12% *SOD1*^*G93A*^ in HT) and MB (10% *WT*; 6% *SOD1*^*G93A*^ in LT; 13% *WT*; 10% *SOD1*^*G93A*^ in HT) (Fig. 2D, F). This indicates that weaker or less dense connections may be labelled in the HT cohort. The targeting of the same regions by different rabies concentrations confirmed the selectivity of the tracing, while dose-dependent redistribution indicated stronger, functionally significant and more sensitive connections within the hypothalamic circuit than in other circuits.

Therefore, MCH neurons are integrated within a broader, brain-wide network, whereas the core circuitry and the most rabies-sensitive projections to MCH are within the hypothalamus. At early-symptomatic disease stages, the overall architecture of MCH large-scale connectivity is maintained, resistant to mutant SOD1 effects.

### Disruption of meso-scale hypothalamic inputs from zona incerta (ZI) to MCH neurons in early-symptomatic ***SOD1***^***G93A***^ mice

We hypothesized that despite the general preservation of MCH networks, disease may impact more functionally relevant inputs from specific subregions. We set to further characterise the integrity of monosynaptic inputs to MCH neurons from brain subregions at a higher, meso-scale resolution. The distribution of inputs within most of these subregions was found to be highly non-uniform (Fig. S5 and S6). In a LT cohort (which favours stronger connections than HT), among 36 hypothalamic subregions where input neurons were found, the majority of inputs originated from the LHA itself, and were preserved in *SOD1*^*G93A*^*;Pmch-Cre* (22% *WT* vs 20% *SOD1*^*G93A*^ in LT) (Fig. 2G). We detected other hypothalamic areas rich in mCherry + neurons—hotspots of MCH inputs, including the zona incerta (ZI), ventromedial hypothalamic nucleus (VMH), tuberal nucleus (TU) and dorsomedial nucleus of hypothalamus (DMH) (Fig. 2I, K; Fig. S5A). The quantification revealed a significantly reduced proportion of ZI-to-MCH inputs in *SOD1*^*G93A*^*;Pmch-Cre* compared to control mice (4% vs 7%, respectively). The inputs from all other hypothalamic nuclei were unchanged in *SOD1*^*G93A*^*;Pmch-Cre* vs *WT;Pmch-Cre mice* (Fig. 2I, K; Fig. S5A). Given the observed dose-dependent effect of the rabies virus on the hypothalamic MCH network we investigated the impact of HT rabies on meso-scale connectivity. As anticipated, the ratio of hotspot inputs from the LHA, VMH, DMH and TU and other nuclei to MCH decreased in comparison to the LT cohort, and to the similar extent in both genotypes (Fig. 2H, J; Fig. S6A). Conversely, the fraction of ZI-to-MCH inputs remained the same as in the LT cohort and significantly lower in *SOD1*^*G93A*^*;Pmch-Cre* than in control mice (3% vs 8%, respectively) (Fig. 2J, L), thus confirming findings in the LT cohort. Furthermore, the inputs from the LPO, MPO, PH and PVH were also unchanged by HT virus (Fig. 2J; Fig. S6A).

In conclusion, the fine-grained mapping of the MCH upstream, hypothalamic network revealed the remarkable disruption of ZI-to-MCH connections associated with *SOD1* transgene expression in ALS mice. Its presence early in the pathogenesis of ALS, before an overt metabolic phenotype and MCH loss, implies an important role of these inputs in MCH dysfunction and degeneration and related metabolic imbalance.

### Extra-hypothalamic meso-scale connectivity of MCH is altered in early-symptomatic ***SOD1***^***G93A***^ mice

**Fig. 3:**
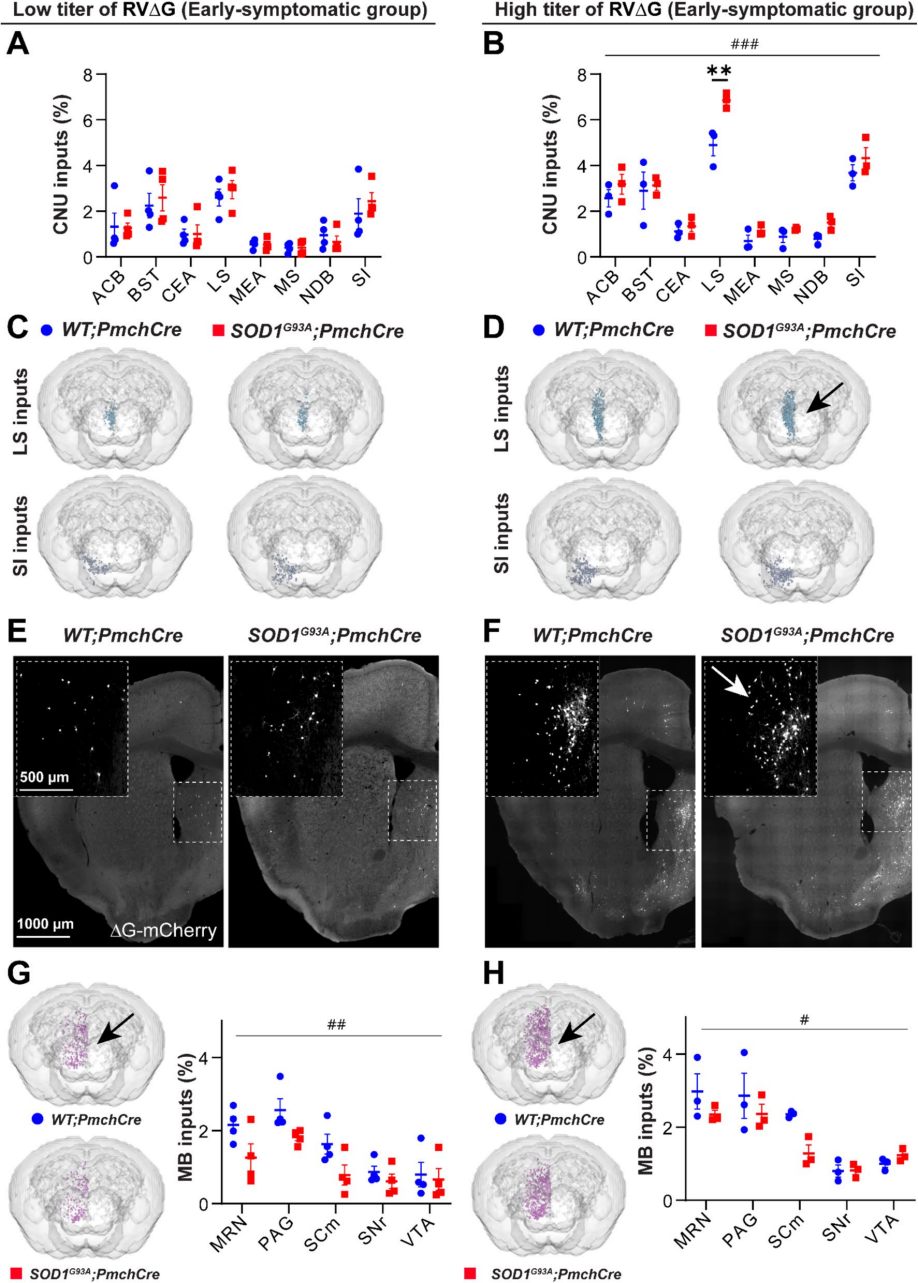
CNU and MB to MCH neurons in early-symptomatic *SOD1*^*G93A*^*;Pmch-Cre* mice. **A-B** The distribution of selected meso-scale inputs from the CNU subregions in the LT **A** and HT **B** cohorts. A significant genotype effect (F1,32 = 13.68, *p* = 0.0008) by 2-way ANOVA and a significant increase in the ratio of inputs from the LS (*p* = 0.003) in pairwise comparison were detected in *SOD1*^*G93A*^*;Pmch-Cre* mice injected with an HT rabies virus **B**. **C-D** 3D reconstructions of inputs from the LS (upper panels) and SI (lower panels) in the LT **C** and HT **D** cohorts. **E-F** Representative images of the rostral coronal brain sections (at Bregma + 0.8 mm) illustrating the location of mCherry + input neurons (white) in the LS in both cohorts: LT **E** and HT **F**. Note the increased density of mCherry + neurons in animals injected with the HT rabies virus **F** and in the LS (white arrow) of *SOD1*^*G93A*^*;Pmch-Cre* (**F**, right panel). **G-H** 3D reconstructions and quantification of MB inputs in the LT **G** and HT **H** cohorts. A significant effect of the *SOD1* transgene on lowering the ratio of inputs from the entire MB (black arrows) and from the selected MB subregions (the quantification graph) was observed in both cohorts: LT (F1,30 = 11.52, *p* = 0.002, **G**) and HT (F1,20 = 4.64, *p* = 0.04, **H**). N = 4 animals per genotype for LT and N = 3 for HT virus concentration. 2-way ANOVA followed by Bonfferoni’s multiple comparisons test, (#) *p*-value < 0.05, (##) *p*-value < 0.01, (###) *p*-value < 0.001 for genotype effect and (**) *p*-value < 0.01 for pairwise comparison. Anatomical abbreviations are based on the ABA, and are fully defined in Supplementary Table 3. Data are displayed as the mean ± SEM of the percentages of neurons found per region/area, with each data point representing a single animal. Scale bars: 1000 μm and 500 μm for insets

Next, we investigated the patterns of distant connections from CNU, CTX and MB to MCH neurons in healthy and ALS mice. Among the 19 CNU subregions containing input neurons, most inputs came from the following subregions in a rank order: lateral septum (LS), the bed nucleus of the stria terminalis (BST), substantia innominata (SI), and nucleus accumbens (ACB), irrespective to genotype or titer of virus (Fig. 3A-F; Fig. S5B; Fig. S6B). No significant difference was detected for projections from any of the subregions between genotypes in the LT cohort (Fig. 3A, C, E). Tracing with HT rabies enhanced the ratio of inputs from the same structures detected with LT and 2-way ANOVA revealed a significant effect of *SOD1* transgene expression (F1,32 = 13.68, *p* = 0.0008) (Fig. 3B, D). Interestingly, the fraction of connections from LS-to-MCH neurons was augmented in *SOD1*^*G93A*^*;Pmch-Cre* compared to *WT;Pmch-Cre* mice (7% vs 5%, respectively), as represented by increased density of retrogradely labelled, mCherry + neurons in LS nuclei (Fig. 3B, D, F). This enhancement was not observed in other CNU subregions projecting to MCH, highlighting a selective increase in LS-to-MCH connectivity. However, this was detected only in the HT cohort.

Among 23 MB areas containing input neurons, most inputs came from midbrain reticular nucleus (MRN), periaqueductal gray (PAG), motor related superior colliculus (SCm), ventral tegmental area (VTA) and substantia nigra (SNr) and this pattern was similar in both rabies titre cohorts (Fig. 3G, H; Fig. S5D; Fig. S6D). The fractions of inputs from most subregions were lower in *SOD1*^*G93A*^*;Pmch-Cre* compared to control mice and a significant effect of *SOD1* mutation was detected by 2-wayANOVA in both rabies titer cohorts (LT: F1,30 = 11.52, *p* = 0.002; HT: F1,20 = 4.64, *p* = 0.04) (Fig. 3G, H).

**Fig. 4:**
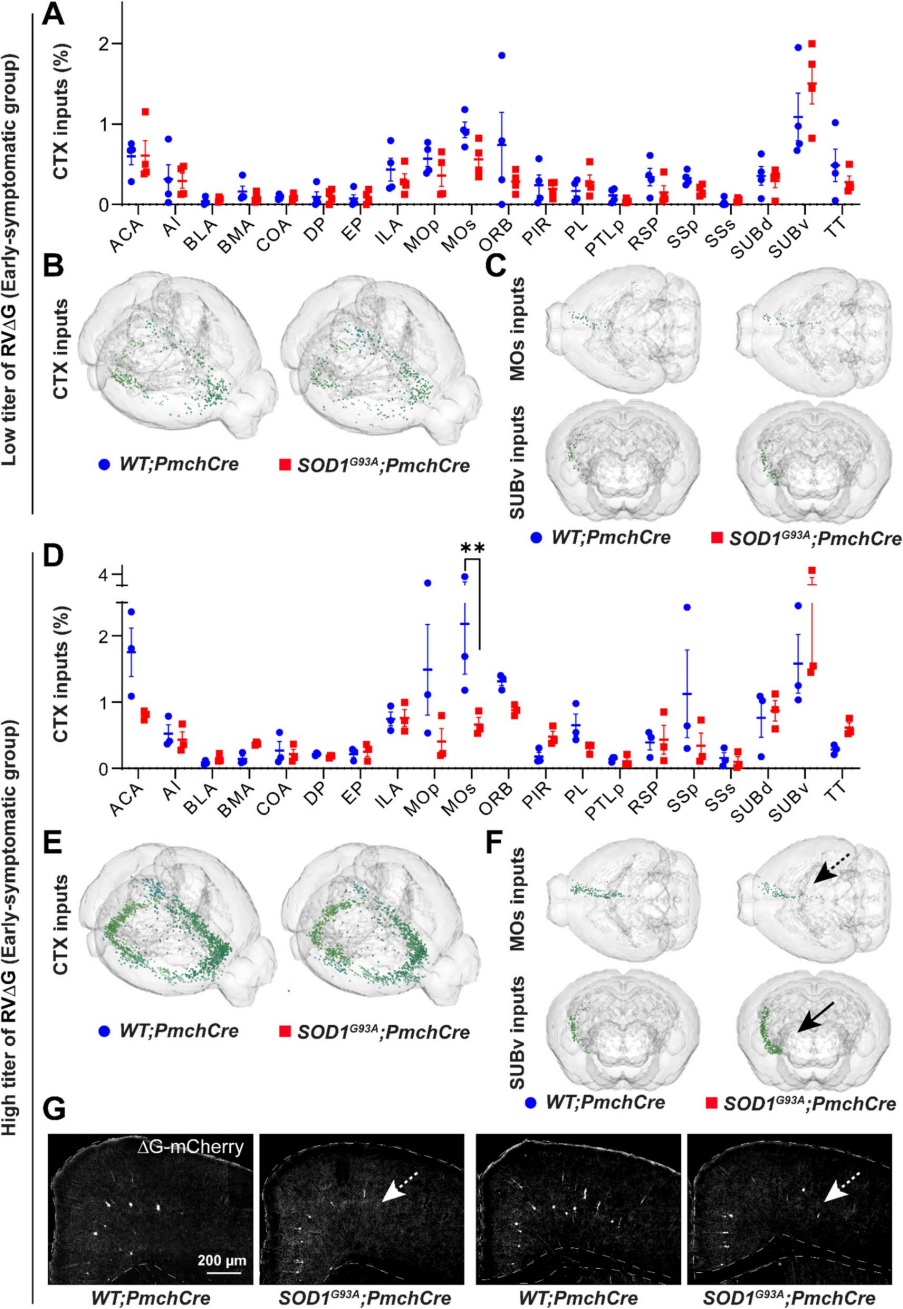
CTX inputs to MCH neurons in early-symptomatic *SOD1*^*G93A*^*;Pmch-Cre* mice. **A** The connectivity pattern of selected meso-scale inputs across the CTX subregions in the LT cohort. A trend for genotype effect (F1,120 = 3.07, *p* = 0.08) towards decreased CTX inputs was identified by 2-way ANOVA in *SOD1*^*G93A*^*;Pmch-Cre* mice. **B-C** 3D reconstructions of inputs from the entire CTX **B** and from the MOs and the SUBv **C**. **D** Connectivity pattern of the CTX subregions in the HT cohort. A trend for genotype effect (F1,80 = 3.43, *p* = 0.07) towards decreased cortical inputs by 2-way ANOVA and a significant decrease in the ratio of inputs from the MOs (*p* = 0.006) in pairwise comparison were identified in *SOD1*^*G93A*^*;Pmch-Cre* mice. **E-F** 3D reconstructions of inputs from the whole CTX **E** and from MOs and SUBv **F**. Note the reduction of inputs from the MOs (black dashed arrow). **G** Representative images of the cortex demonstrating a reduction in the density of mCherry + input neurons (white dashed arrows) located in the motor cortex in the HT cohort. N = 4 animals per genotype for LT and N = 3 for HT virus concentration. 2-way ANOVA followed by Bonfferoni’s multiple comparisons test, (**) *p*-value < 0.01 for pairwise comparison. Anatomical abbreviations are based on the ABA, and are fully defined in Supplementary Table 3. Data are displayed as the mean ± SEM of neuron percentages found per region/area, with each data point representing a single animal. Scale bars: 200 μm

Among 30 CTX areas containing input neurons, the largest number were found in the ventral subiculum (SUBv), motor cortex (MO), orbital area (ORB), anterior cingulate area (ACA) and infralimbic area (ILA). This pattern was consistent with our previous report [5], and was similar in both titre cohorts (Fig. 4A-F; Fig. S5C; Fig. S6C). A significantly decreased ratio of inputs from secondary motor cortex (MOs) was detected *SOD1*^*G93A*^*;Pmch-Cre* compared to *WT;Pmch-Cre* mice only in HT group (0.7% vs 2%, respectively) (Fig. 4D, F, G). While the inputs from other cortical subregions remain unchanged between genotypes a strong trend for the effect of mutant *SOD1* was noticed by 2-way ANOVA in both rabies titer cohorts (LT: F1,120 = 3.07, *p* = 0.08; HT: F1,80 = 3.43, *p* = 0.07) (Fig. 4A, D).

Overall, these results indicate that early in disease structural integrity of upstream, extra-hypothalamic MCH network is affected by *SOD1* transgene although to a much lesser extent than hypothalamic network and only emerges when the long, distant and likely the least sensitive projections to retrograde tracing are targeted by increased rabies titer. Notably, projections from MOs-to-MCH are significantly reduced while projections from LS-to-MCH are increased, suggesting subtle connectivity alterations detectable only under high-coverage conditions.

### Hypothalamic connectivity of MCH neurons declines with disease progression in late-symptomatic ***SOD1***^***G93A***^ mice

**Fig. 5:**
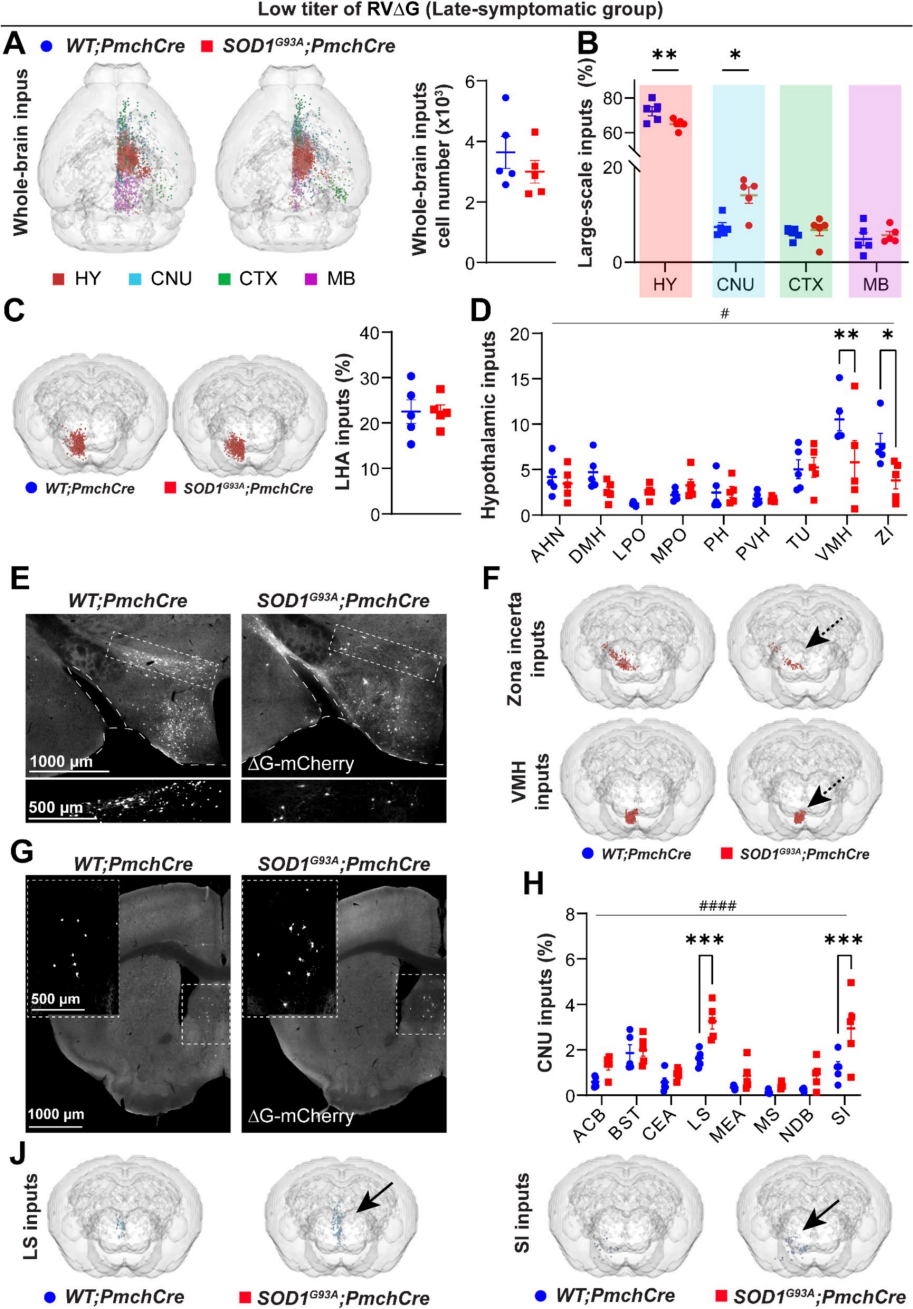
Upstream MCH network in late-symptomatic *SOD1*^*G93A*^*;Pmch-Cre* mice. **A** Representative *WholeBrain* 3D reconstructions illustrate the location of neurons, projecting to the MCH, within large-scale brain regions in *WT;Pmch-Cre* and *SOD1*^*G93A*^*;Pmch-Cre* mice. The quantification graph shows the total sum of monosynaptic inputs from the whole brain to the MCH in *WT;Pmch-Cre* (blue) and *SOD1*^*G93A*^*;Pmch-Cre* (red) mice. No significant difference was observed between the two genotypes. **B** The quantification graph presents the distribution pattern of inputs across the large-scale brain regions. The ratio of HY inputs was significantly reduced (*p* = 0.005) and the ratio of CNU inputs was significantly enhanced (*p* = 0.01) in *SOD1*^*G93A*^*;Pmch-Cre* compared to control mice. **C** 3D reconstructions and quantification of LHA inputs were comparable between the two genotypes. **D** Distribution of inputs across the selected meso-scale HY areas. A significant genotype effect (F1,72 = 4.99, *p* = 0.03) towards reduced inputs across areas, and a significant reduction of the ratio of projections from the ZI (*p* = 0.03) and from the VMH (*p* = 0.006) were detected in *SOD1*^*G93A*^*;Pmch-Cre* mice. **E** Representative images of the hypothalamus (at Bregma −1.3) showing the location of mCherry + input neurons (white). The insets depict a loss of mCherry + input neurons in the ZI in *SOD1*^*G93A*^*;Pmch-Cre* mice(right panel). **F** 3D reconstructions represent inputs from the entire ZI (upper panels) and VMH (lower panels), which are reduced in *SOD1*^*G93A*^*;Pmch-Cre* mice (black dashed arrows). **G** Representative images of the rostral coronal brain section (at Bregma + 0.8) illustrating the location of mCherry + input neurons (white) in the LS. **H** Distribution of selected meso-scale inputs across the CNU subregions. A significant genotype effect (F1,64 = 31.57, *p* < 0.0001) and a significant increase in the ratio of inputs from the LS (*p* = 0.0005) and from the SI (*p* = 0.0002) were detected in *SOD1*^*G93A*^*;Pmch-Cre* mice. **J** 3D reconstructions depict inputs from the entire LS (left panels) and SI (right panels), which are increased in *SOD1*^*G93A*^*;Pmch-Cre* (black arrows). N = 5 animals per genotype. Two-tailed unpaired Student’s t-test **A, C** for comparison between two genotypes and 2-way ANOVA followed by Bonfferoni’s multiple comparisons test **B, D, F**, (#) *p*-value < 0.05, (####) *p*-value < 0.0001 for genotype effect and (*) *p*-value < 0.05, (**) *p*-value < 0.01, (***) *p*-value < 0.001 for pairwise comparison. Data are mean ± SEM of neuron percentages found per region/area, except for absolute values (**A**), with each data point representing a single animal. Scale bars: 1000 μm (for lower magnification images) and 500 μm (for higher magnification images)

Next, we investigated the impact of ALS disease progression on MCH network. To this end, we conducted an analysis of the MCH connectome in late-symptomatic mice that had been injected with LT rabies virus (P75-AAV inj.; P105-RV∆G inj.; P113-sacrifice). This approach was selected due to the fact that LT targeted the core circuitry in a more specific manner, despite the inherent risk of under-representing broader connectivity. The total number of input neurons from the whole-brain was comparable between genotypes (Fig. 5A). However, the analysis of the distribution of inputs among large-scale regions revealed a significant decline in the ratio of the inputs from HY and a significant increase in the ratio of inputs from CNU in *SOD1*^*G93A*^ mice compared to *WT;Pmch-Cre* mice (64% vs 72%-HY; 14% vs 7%-CNU, respectively) (Fig. 5B). The decline in hypothalamic connectivity observed at the large-scale was not attributable to the loss of the major input fraction from the LHA (Fig. 5C). This occurred as a consequence of a significantly lower fraction of inputs from ZI and newly detected a significant reduction in VMH-originating inputs to MCH neurons in *SOD1*^*G93A*^ mice, as revealed by analysis at meso-scale resolution (4% vs 8%-ZI; 6% vs 11%-VMH, respectively) (Fig. 5D, E, F; Fig. S7A). While inputs from other HY nuclei remain unaltered, a genotype effect was identified by 2-way ANOVA (F1,72 = 4.99, *p* = 0.03) (Fig. 5D). Thus, disruption of MCH network expands during the late-symptomatic stage, correlating with systemic metabolic imbalances and MCH degeneration.

A quantitative meso-scale analysis of extra-hypothalamic projections showed a significant effect of the *SOD1* mutation on the distribution of labelled inputs from distinct CNU subregions (Fig. 5H; Fig. S7B, 2-way ANOVA (F1,64 = 31.57, *p* < 0.0001)), including a significant increase in the proportion of inputs from LS-to-MCH neurons (Fig. 5G, H), accompanied by increased inputs from the SI at late-symptomatic *SOD1*^*G93A*^ mice in comparison to age-matched controls (3.2% vs 1.6%-LS; 2.9% vs 1.2%-SI, respectively) (Fig. 5H, J).

The projections from the MB and CTX subregions to MCH neurons remain unchanged between the genotypes (Fig. S7C, D). Of note, the lower total number of input neurons in late-symptomatic mice in comparison to the numbers in early-symptomatic mice suggests a decline in retrograde tracing efficiency in aged mice, regardless of genotype.

Overall, mutant *SOD1* leads to a decrease in upstream large-scale HY-to-MCH connectivity and a dynamic reorganization of the CNU-to-MCH network. The impairment of the meso-scale network architecture, loss of ZI-to-MCH connections, persists in the late disease stage and it is accompanied by an additional loss of VMH inputs, indicating a progressive disintegration of the HY network and a particular vulnerability of the ZI. In parallel, early increased projections from the LS followed by projections from the SI may reflect adaptive remodelling of the CNU network. MO connections appear to be affected only at high rabies titers, suggesting early but subtle disconnection. These region-specific changes suggest a potential shift in MCH regulation from HY to CNU circuits.

### ZI-to-MCH projections are DAergic and GABAergic and loss of DAergic neurons contributes to the network disruption

**Fig. 6:**
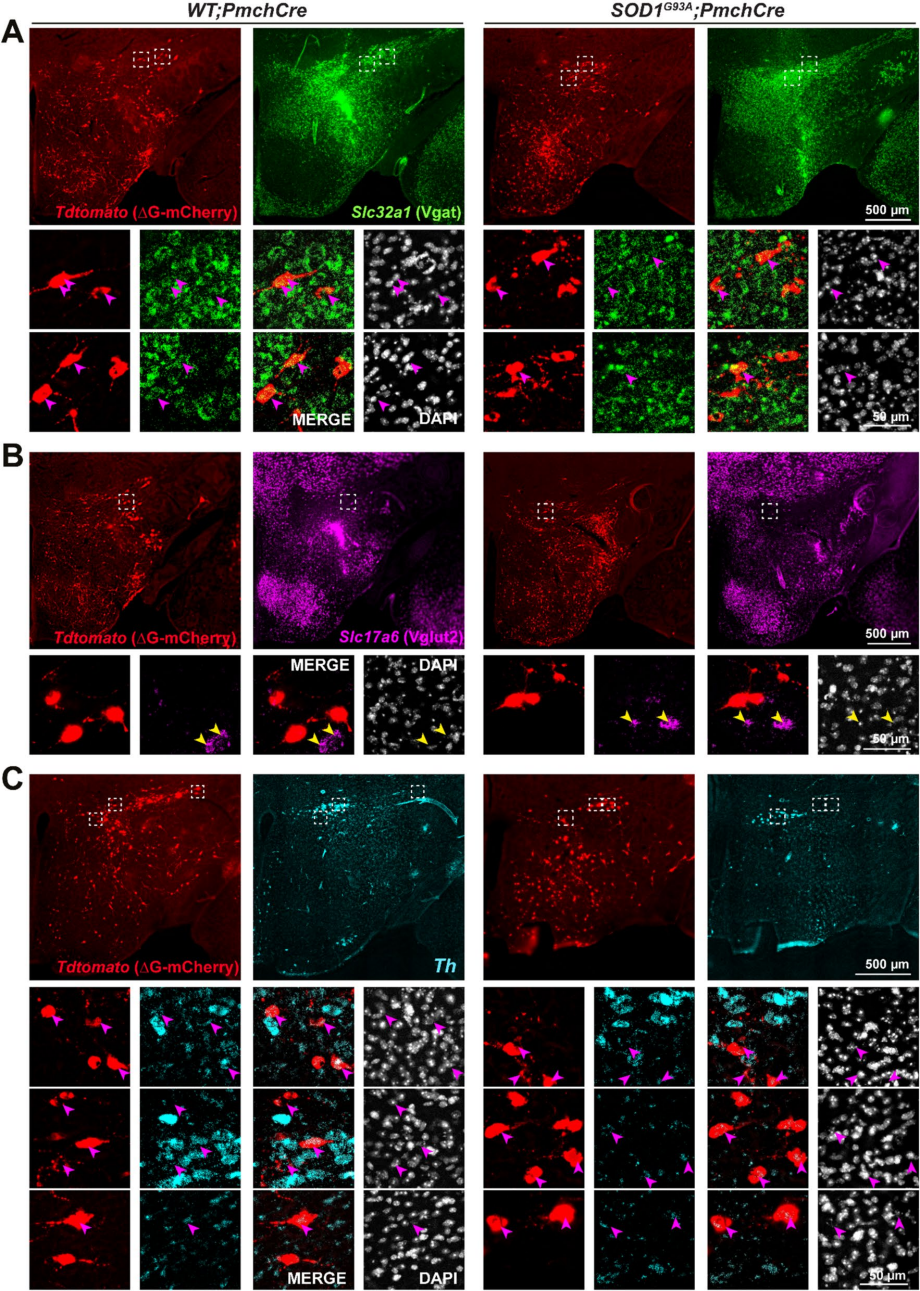
GABAergic and DAergic neurochemical identity of ZI-to-MCH projections. **A-C** Representative images of the hypothalamus (overview) and the ZI (enlarge insets–white dashed line square) showing the spatial distribution of input neurons from ZI co-expressing mRNA encoding *tdTomato* (red) and **A** *Slc32a1*, mRNA encoding VGAT (green) (white arrowheads), or **B** *Slc17a6* mRNA encoding VGlut2 (magenta), or **C** *Th* mRNA which encoding tyrosine hydroxylase (cyan) (yellow arrowheads), all detected by a single-molecule mRNA in situ hybridization. Representative examples from three * WT;Pmch-Cre* and three *SOD1*^*G93A*^*;Pmch-Cre* brains. Scale bars: 500 μm (for lower magnification images) and 50 μm (for higher magnification images)

The disruption of the ZI-to-MCH network was consistently replicated in ALS mice in our tracing study, thereby suggesting the important role of this network in triggering MCH vulnerability. We reasoned that disease-related deficient synaptic signalling may be the leading mechanism. Indeed, it has been demonstrated that MCH could respond to a wide variety of presynaptic signalling molecules, encompassing transmitters, modulators and peptides [33]. To ascertain the neurochemical identity of neurons projecting from ZI-to-MCH we again exploited the multiplexed RNAScope assay. This was conducted in the early-symptomatic HT cohort (P88-sacrifice), given to the highest density of retrogradely traced neurons. Rabies-infected neurons were visualized by a strong expression of mRNA encoding *tdTomato* (similar to mCherry reporter). A substantial portion of ZI *tdTomato* + neurons in both *WT;Pmch-Cre* and *SOD1*^*G93A*^*;Pmch-Cre* mice co-expressed *Slc32a1* mRNA encoding the gamma-aminobutyric acid (GABA) vesicular transporter (VGAT), and revealed their GABAergic signalling (Fig. 6A). In contrast, the population of *tdTomato* + neurons in the ZI rarely expressed *Slc17a6* mRNA, which guides the synthesis of vesicular glutamate transporter (VGlut2), and characterizes glutamatergic excitatory neurons (Fig. 6B). Unexpectedly, in the ZI we detected a discrete population of *tdTomato* + neurons that co-expressed low levels of tyrosine hydroxylase (*Th*) mRNA (Fig. 6C). It has been shown that the *Th* + population in ZI is exclusively dopaminergic (DAergic) and not noradrenergic, as for lacking the expression of dopamine β-hydroxylase (DBH), and that these DAergic neurons always co-transmit GABA [57]. We further confirmed that ZI/DAergic neurons are projecting to MCH neurons as evidenced by the co-localization of TH + fibre endings and dopamine transporter—DAT + fibres/puncta with MCH + cell bodies or dendrites in both *WT* and *SOD1*^*G93A*^ mice at P100 (late-symptomatic mice) (Fig. S8A–C). As well, ZI/DAergic neurons received projections from MCH neurons (Fig. S8D, E), demonstrating bidirectional connections.

**Fig. 7:**
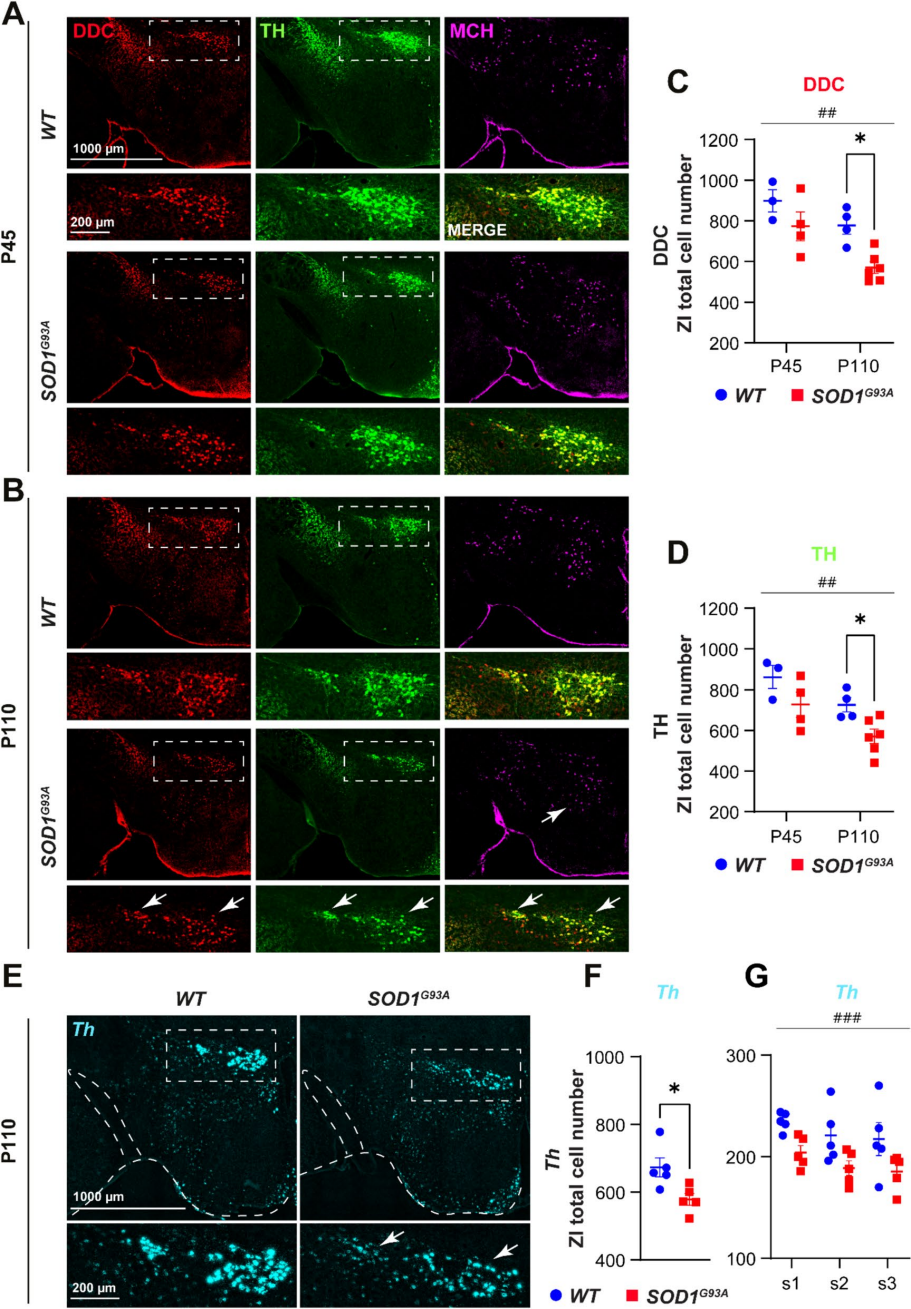
Loss of ZI/DAergic neurons in the late-symptomatic disease stage. **A **and** B **Representative images of coronal hypothalamic sections (overview) showing the distribution of DAergic neurons in the ZI (enlarged insets–white dashed line square) co-expressing the enzymes for dopamine synthesis DDC (red) and TH (green) in close proximity to MCH neurons (magenta) in *WT* (upper panels) and *SOD1*^*G93A*^ (lower panels) mice at P45 **A** and at P110 **B**. Loss of DDC + /TH + neurons and MCH neurons is evident in *SOD1*^*G93A*^ mice at P110 (white arrows). **C** and** D** The graphs present the total number of neurons quantified on six rostro-caudal sections in *WT* (blue) and *SOD1*^*G93A*^ (red) mice at P45 and at P110. DDC + (*p *= 0.01) **C** and TH + (*p* = 0.04) **D** neurons are significantly reduced in *SOD1*^*G93A*^ mice at P110 – late-symptomatic disease stage in pairwise comparison, with the significant 2-way ANOVA genotype effect for DDC + (F1,13 = 11.58, *p* = 0.005) **C** and for TH + neurons (F1,13 = 9.43, *p* = 0.009) **D**. N = 3–6 animals per genotype. **E** Representative images showing neurons expressing Th mRNA (cyan) in the HY and ZI (insets). **F** and** G** Quantification of *Th* + neurons on three sections confirmed a significant reduction (*p* = 0.02) in a second cohort of P110 *SOD1*^*G93A*^ mice **F** and the significant 2-way ANOVA genotype effect (F1,24 = 15.10, *p* = 0.0007) **G**. N = 5 animals per genotype. 2-way ANOVA followed by Bonfferoni’s multiple comparisons test, (##) *p*-value < 0.01, (###) *p*-value < 0.001 for genotype effect and (*) *p*-value < 0.05 for pairwise comparison. Two-tailed unpaired Student’s t-test **F**. Data are displayed as the mean ± SEM, with each data point representing a single animal. Scale bars: 1000 μm (for lower magnification images) and 200 μm (for higher magnification images)

As early synaptic damage often precedes neuronal death in ALS and other neurodegenerative diseases, we investigated whether impaired ZI-to-MCH connectivity is caused solely by synaptic loss or whether it also involves the degeneration of ZI/DAergic neurons. To this aim DAergic population was assessed in independent cohorts of mice at P45 (pre-symptomatic) and P110 (late-symptomatic) by immunolabelling against the enzymes critical in DA synthesis TH and DOPA-decarboxylase (DDC). Whereas the quantified TH + and DDC + neurons were comparable between *WT* and *SOD1*^*G93A*^ mice at P45 (Fig. 7A, C, D), at P110 *SOD1*^*G93A*^ mice displayed a significant decline of DAergic neurons within the ZI (Fig. 7B, C, D). To eliminate the possibility that the observed DAergic loss could be attributed to a deficiency in protein synthesis, in situ hybridization for *Th* was performed on an independent set of samples at P110 and the quantification confirmed a significant reduction in *Th* + neurons (Fig. 7E, F, G), indicating late-onset DAergic degeneration. Additionally, we observed more *Th* expressing neurons than those showing TH immunoreactivity, which has been noted before in the hypothalamus [8]. The loss of DAergic neurons was selective to ZI as the *Th* + neurons in the LHA and ARH were comparable between *WT* and *SOD1*^*G93A*^ mice (Fig. S9A–D).

Taken together, these findings imply that GABAergic and DAergic presynaptic inputs onto MCH neurons are particularly susceptible to ALS and *SOD1* mutations and most likely the main contributors to ZI-to-MCH network disruption starting at the synaptic level.

### ALS-related molecular and cellular pathways are enriched in ZI and in DAergic neurons

**Fig. 8:**
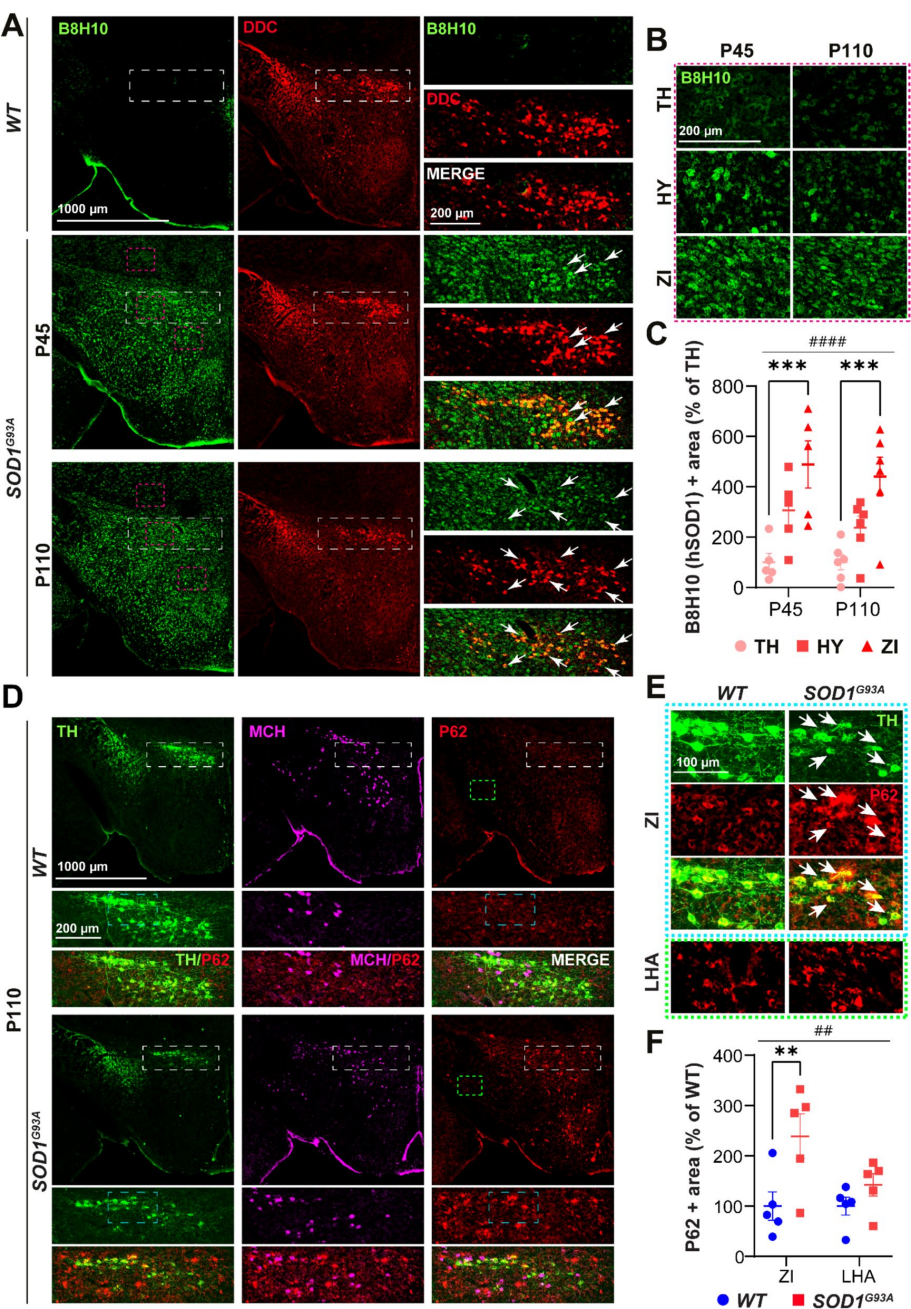
Disease burden is enhanced in ZI and in DAergic neurons in pre-symptomatic *SOD1*^*G93A*^ mice. **A.** Representative images of coronal hypothalamic sections (overview) and ZI (enlarged insets–white dashed line square) showing the absence of misfolded SOD1 (B8H10, green) in *WT* (upper panel) and its high expression in *SOD1*^*G93A*^ (lower panels) mice at P45 and at P110, co-localizing with DDC + neurons (red) (white arrows). **B** Enlarged insets (red dashed line square) illustrate the difference in density of misfSOD1 + neurons and in misfSOD1 expression levels in different regions: the thalamus (TH), the HY and the ZI (red dashed line square). **C** The quantification graph demonstrates a significant region effect by 2-way ANOVA (F2,27 = 17.86, *p* =  < 0.0001) due to a significant increase in the misfSOD1 + area in the ZI compared to the TH (*p* = 0.0006 at P45; *p* = 0.0009 at P110) and a trend compared to the HY (*p* = 0.1 at P45; *p* = 0.06 at P110). **D** Immunofluorescence images representing P62/SQSTM1 (red), TH (green) and MCH (magenta) immunoreactivity in the hypothalamus (overview), the ZI (enlarged insets–white dashed line square) and the LHA (green dashed line square) at P110 *WT* (upper panel) and *SOD1*^*G93A*^ (lower panels) mice. **E** Enlarged insets (cyan dashed line square) depict P62/SQSTM1 inclusions in the ZI and in DAergic TH + neurons (white arrows) in *SOD1*^*G93A*^ mice (right panel), which are absent from the LHA. **F** The quantification graph demonstrates a significant increase of P62 + area in the ZI in *SOD1*.^*G93A*^ compared to *WT* mice (*p* = 0.009 in pairwise comparison and the genotype effect by 2-way ANOVA (F1,16 = 9.15, *p* = 0.008)). N = 5 animals per genotype. 2-way ANOVA followed by Bonfferoni’s multiple comparisons test, (##) *p*-value < 0.01, (####) *p*-value < 0.0001 for region and genotype effect and (**) *p*-value < 0.01, (***) *p*-value < 0.001 for pairwise comparison. Data are displayed as the mean ± SEM, with each data point representing a single animal. Scale bars: 1000 μm (for lower magnification images) and 200 μm (for higher magnification images)

In order to gain a deeper insight into this regional, ZI vulnerability we examined the pathways that are commonly implicated in ALS pathophysiology. We evaluated the burden of misfolded SOD1 (misfSOD1) using an antibody specific to this toxic form of SOD1 (B8H10). As anticipated, immunohistochemical analysis revealed elevated levels of misfSOD1 in *SOD1*^*G93A*^ compared to *WT* mice, irrespective of age (Fig. 8A). An increase in the density of misfSOD1 + neurons was observed within the ZI in contrast to surrounding areas where levels were comparatively low (Fig. 8B). DAergic neurons displayed elevated burden of misfSOD1, as evidenced by the co-localisation of misfSOD1 in DDC + neurons. Image-based quantification demonstrated a statistically significant increase in misfSOD1 + area in the ZI compared to the other hypothalamic and thalamic regions (Fig. 8C), present as early as P45 (pre-symptomatic), and to a similar extent as in the canonically affected layer 5 of the motor cortex (data not shown). Given that misfolded SOD1 is associated with impaired autophagic clearance [16] we monitored the levels and distribution of autophagy marker P62/SQSTM1, previously reported to accumulate in LHA of *Sod1*^*G86R*^ mice [31]. Immunofluorescent staining revealed a robust P62 immunoreactivity and inclusions within the ZI of *SOD1*^*G93A*^ mice at P110 (late-symptomatic), in contrast to a minimal presence of P62 in the ZI of *WT* mice (Fig. 8D). Accumulation of P62 inclusions was observed in the extracellular matrix and within neuronal cell bodies, including DAergic, TH + /P62 + neurons (Fig. 8E). The quantification demonstrated a statistically significant increase in P62 + area in the ZI compared to the neighbouring region of LHA (Fig. 8F), indicating the presence of targeted protein aggregation, autophagic and proteostatic vulnerability in this region.

Finally, we investigated the expression of markers associated with degeneration and inflammation, namely the astrogliosis marker GFAP, and the microgliosis marker IBA1 [86] (Fig. S10A, B). Quantification of the GFAP-immunoreactive area demonstrated a statistically significant enrichment in astrocyte coverage of the entire hypothalamus at P110 (Fig. S10C), and specifically of the ZI at P45 and at P110 (Fig. S10D), in *SOD1*^*G93A*^ mice compared to their age-matched *WT* littermates, highlighting enhanced astrocytic activity. Astrocytes were observed in close proximity to DAergic, TH + neurons. Surprisingly, no significant difference in the area occupied by IBA1 + microglia was detected in the hypothalamus nor in the ZI between genotypes (Fig. S10A, B, E, F), indicating the existence of a unique pattern of neuroinflammatory response in ALS hypothalamus.

These results suggest that the ZI is a region of enhanced vulnerability in ALS, in particular DAergic neurons, displaying notable alterations across multiple ALS-related pathways associated with neurodegeneration. The presence in early disease stages underscores their potential mechanistic role in ALS-related ZI-to-MCH network disruption, MCH and metabolic dysregulation and suggests that the ZI may be an initially affected hypothalamic region.

### ALS-affected motor cortex neurons directly target vulnerable MCH and DAergic neurons

**Fig. 9:**
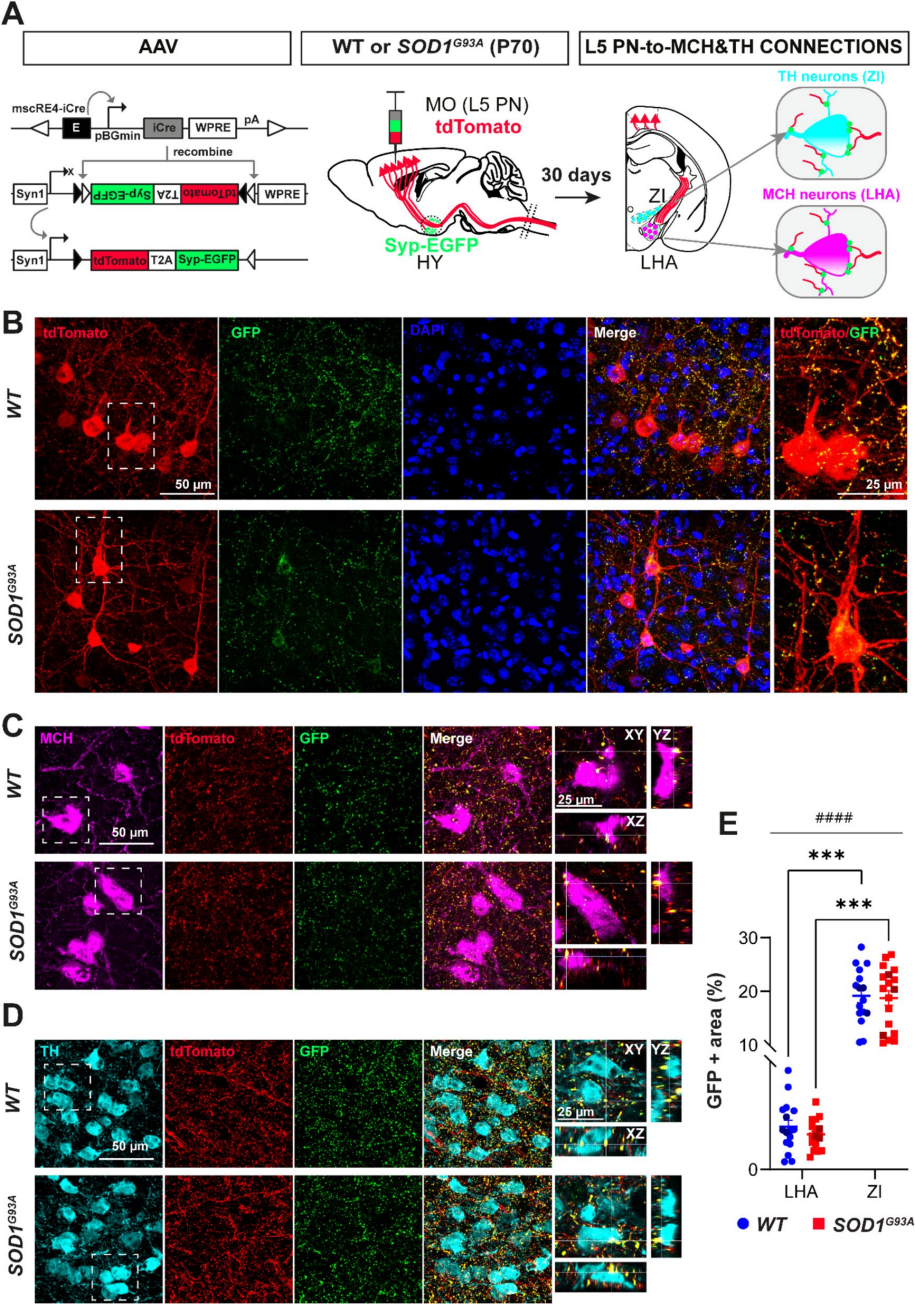
Neuronal projections from L5 PN in the motor cortex to hypothalamic MCH and ZI/DAergic neurons. **A** The scheme outlines the experimental workflow for the selective anterograde tracing of the motor cortex layer 5 projection neurons (L5 PN) and labelling their synaptic contacts using cell-type-specific viral tools injected into the motor cortex of P70 *WT* and *SOD1*^*G93A*^ mice. The virus containing: a putative enhancer, mouse single-cell regulatory elements (mscRE4) and beta-globin minimal promoter (pBGmin) driving expression of a mouse codon-optimized Cre (iCre), a short woodchuck hepatitis virus post-transcriptional regulatory element (WPRE), and polyadenylation site (pA), was co-delivered with the virus expressing the fluorescent proteins tdTomato in the neuronal cytoplasm and SypEGFP in the presynaptic compartment under the synapsin 1 promoter (Syn1) and in Cre-dependent manner. After 30 days, connections were visualized by co-localization of the fluorescent proteins with immunolabeled neurons: MCH + in LHA and TH + in ZI. **B** Representative confocal images of the MO depicting L5 PN neurons expressing iCre and tdTomato (red) in the cytoplasm, dendrites and axon, together with GFP + puncta showing synaptic localization (green). **C-D** Representative confocal images of the LHA depicting MCH neurons (magenta) **C** and images of the ZI showing TH + neurons (cyan) **D** surrounded by dense tdTomato + fibres (red) and GFP + puncta (green). The enlarged inset (white dashed line square) and the orthogonal XZ and YZ views illustrate the co-localization of the reporter proteins with the cell-body of an MCH + or TH + neuron. e. The quantification plot shows a significantly higher percentage of GFP + area in ZI compared to area occupied in LHA in both genotypes. N = 3 animals per genotype. 2-way ANOVA followed by Bonfferoni’s multiple comparisons test, (####) *p*-value < 0.0001, for area effect, and (***) *p*-value = 0.0006 for pairwise comparison. Data are presented as the mean ± SEM and each dark coloured point represents a single animal. Scale bars: 1000 μm and 200 μm (for lower and for higher magnification images) and 50 μm for confocal images

Misfolded SOD1 was present early on in the ZI, similar as in the motor cortex (MO) which is the canonically affected region in ALS, in particular the UMN, a population of the corticofugal cortical layer 5 long projection neurons (L5 PN). According to the corticofugal hypothesis, the MO is considered the initial site of disease onset, with the disease pathology subsequently spreading to neurons in distant regions along direct connections [11]. As we identified direct projections from the MO to vulnerable MCH neurons among the cortical inputs, we used an anterograde mapping strategy, to confirm the accuracy of these connections to MCH neurons and to test whether L5 PN also project to degenerating DAergic neurons. To map L5 PN projections, *WT* and *SOD1*^*G93A*^ animals at P70 were co-injected with the two AAVs in the MO, the first vector encoding for Cre recombinase under the enhancer and under the mini-promoter specific for L5 PN, and the second vector encoding, in a Cre-dependent manner, the expression of the tdTomato reporter in the cytoplasm and of the SypEGFP tag in fibre endings and at synapses to label all the presynaptic contacts originating from the target (Fig. 9A; Fig. S11A, B). Thirty days later (P100-late-symptomatic), the brains were dissected and connections between cortical L5 PN and hypothalamic MCH or DAergic neurons were visualized as green puncta (SypEGFP) along MCH + or TH + dendrites and cell bodies (Fig. 9A). We detected that the vast majority of tdTomato + neurons were localized within layer 5 and some in layer 6, while they were almost completely absent from other cortical layers in both genotypes (Fig. 9B; Fig. S11C). The distribution of tdTomato + fibre tracts across brain sections in rostro-caudal direction replicated that of the pyramidal tract further confirming the selective targeting of the L5 PN (Fig. S11A, B).

In the hypothalamus, and more specifically in the LHA and ZI where MCH and DAergic neurons respectively reside, tdTomato + fibres and co-localized GFP + puncta were observed (Fig. 9C, D; Fig. S12A, B). Quantification of the percentage of the area occupied by GFP + synaptic puncta surrounding MCH neurons in LHA and TH neurons in ZI was comparable between genotypes although slightly lower in *SOD1*^*G93A*^ than in *WT* LHA (approximately −18%), and in line with subtle loss of MO-to-MCH inputs detected by retrograde HT rabies tracing (Fig. S12C, D). Interestingly, ZI receives more robust inputs from the L5 PN compared to LHA in both *WT* and *SOD1*^*G93A*^ mice (Fig. 9E), revealing their distinct connectivity patterns as well as a circuit basis for disease propagation. Since different neuropeptidergic neurons are present in the LHA as well as in the ZI, we further verified that the projections from the L5 PN form the close contacts with MCH and TH neurons as evidenced by the co-localization of tdTomato + fibre endings and GFP + puncta with MCH + or with TH + neurons, either with their cell bodies or dendrites (Fig. 9C, D, orthogonal XZ and YZ views). Each examined MCH + or TH + neuron contained at least one connection (approximately between 3–8 contacts per neuron).

Together, these findings revealed direct synaptic inputs from L5 PN to MCH and DAergic neurons, forming a broader vulnerable network that links ALS-affected motor and metabolic regions. Preferential motor input to ZI over LHA suggests a potential and facilitated spread of ALS pathology from the motor cortex, thereby contributing to the early and amplified vulnerability of ZI.

### The vulnerability of DAergic neurons reflects the body-weight phenotype across ALS murine models

**Fig. 10:**
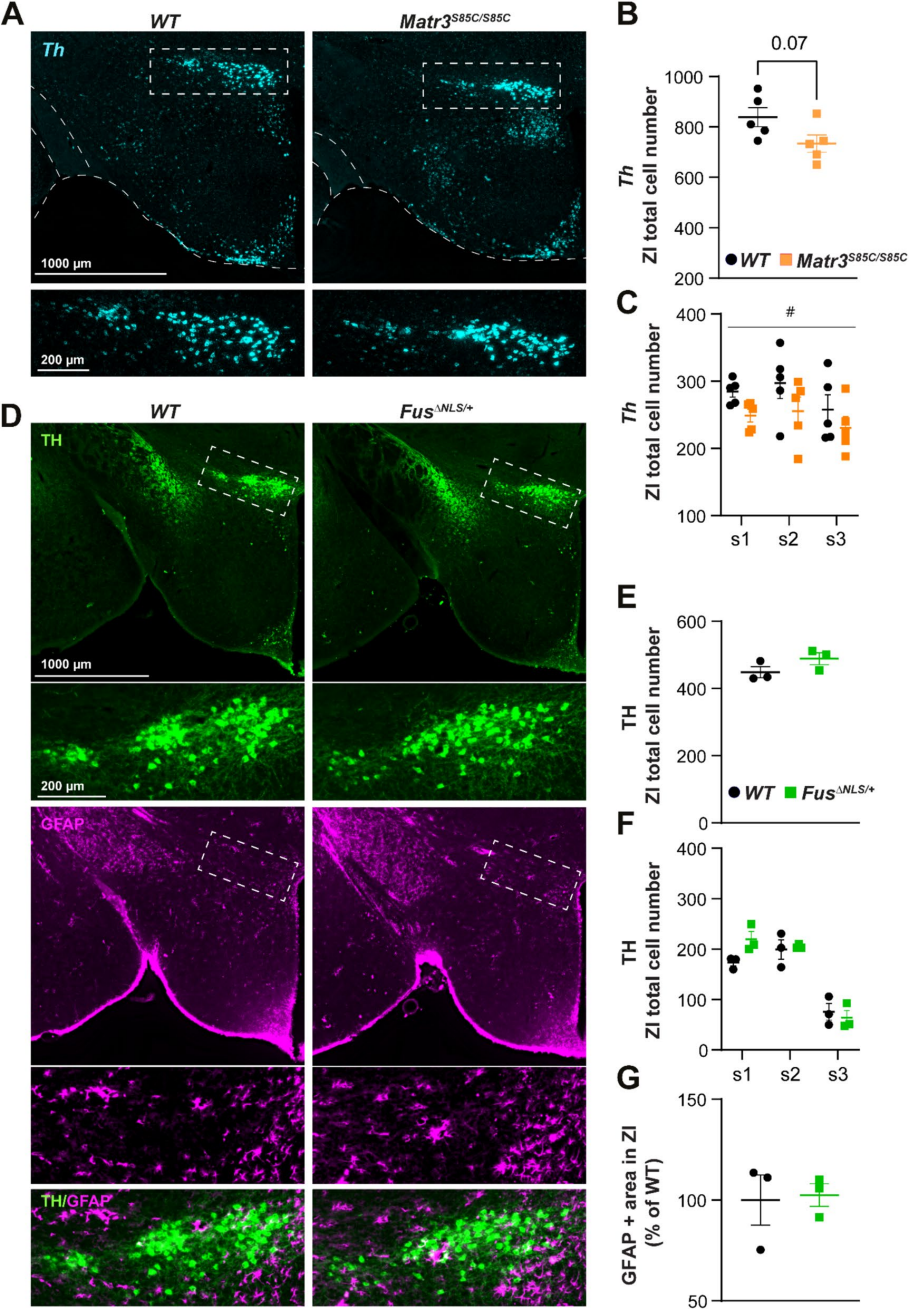
ZI/DAergic neurons are lost in *Matr3*^*S85C/S85C*^ mice, but are preserved in *Fus*^*∆NLS/*+^ mice. **A** Representative images showing neurons expressing *Th* mRNA (cyan) in the hypothalamus (overview) and in the ZI (enlarged insets within white dashed line square) in 50–60-week-old *WT* (left panels) and *Matr3*^*S85C/S85C*^ mice (right panels). **B-C** Quantification of *Th* + neurons in three sections showing a strong trend towards a reduction in *Th* + neurons (*p* = 0.07) in *Matr3*^*S85C/S85C*^ mice (black) compared to their *WT* littermates (orange) **B** and the significant genotype effect 2-way ANOVA (F1,24 = 5.82, *p* = 0.02) **C**. **D** Representative images of coronal hypothalamic sections (overview) showing the distribution of dopaminergic neurons expressing TH (green) and the distribution of surrounding GFAP + astrocytes (magenta) in the ZI (enlarged insets–white dashed line square in 12-month-old *WT* (upper panels) and *Fus*^*∆NLS/*+^ (lower panels) mice. The number of TH + neurons is similar in *Fus*^*∆NLS/*+^ mice and their *WT* littermates. **E–F** The graphs show the total number of neurons quantified on three sections **E** and per section **F** in *WT* (black) and *Fus*^*∆NLS/*+^ (green) mice. **G** Quantification plot shows no difference in the percentage of GFAP + area throughout the ZI in *Fus*^*∆NLS/*+^ compared to *WT* littermates. N = 5 animals per genotype for *Matr3*^*S85C/S85C*^ mice, and N = 3 animals per genotype for *Fus*^*∆NLS/*+^ (green) mice. Two-tailed unpaired Student’s t-test in **B, E, G** 2-way ANOVA followed by Bonfferoni’s multiple comparisons test **C, F**, (#) *p*-value < 0.05, for genotype effect. Data are presented as the mean ± SEM, with each data point representing a single animal. Scale bars: 1000 μm (for lower magnification images) and 200 μm (for higher magnification images)

Finally, to gain insight into the potential relevance of DAergic degeneration for ALS-associated metabolic dysregulation, we evaluated this neuronal population in two additional ALS models, which present distinct body-weight profiles. Namely, we investigated 50–60 -weeks-old homozygous *Matr3*^*S85C/S85C*^ mice, which cease to gain weight at 10 weeks of age [39]. Consistent with their body-weight trajectory, we observed a strong trend (*p* = 0.07) towards a decreased number of *Th* + positive neurons in *Matr3*^*S85C/S85C*^ mice compared to their wild-type littermates (Fig. 10A–C). This reduction in ZI DAergic neurons aligns with the weight phenotype observed in this model, supporting their role in metabolic dysregulation.

In contrast, 12-month-old *Fus*^*∆NLS/*+^ mice, which largely remain normal weight despite MCH loss [7, 68], showed preservation of the TH + population and an absence of astrocyte activation (Fig. 10D–G). The maintenance of ZI DAergic neurons in *Fus*^*∆NLS/*+^ mice suggests a potential neuroprotective mechanism underlying their relative metabolic stability. Moreover, the contrasting effects of *Fus* and *SOD1* mutations on MCH and TH neurons imply differential mutant-gene toxicity and mutation-specific vulnerabilities within hypothalamic circuits.

Taken together, these findings suggest that the loss of ZI DAergic neurons underlies weight loss observed across distinct genetic models, and may contribute to metabolic dysregulation associated with ALS, aligning molecular pathology with functional outcomes.

## Discussion

**Fig. 11:**
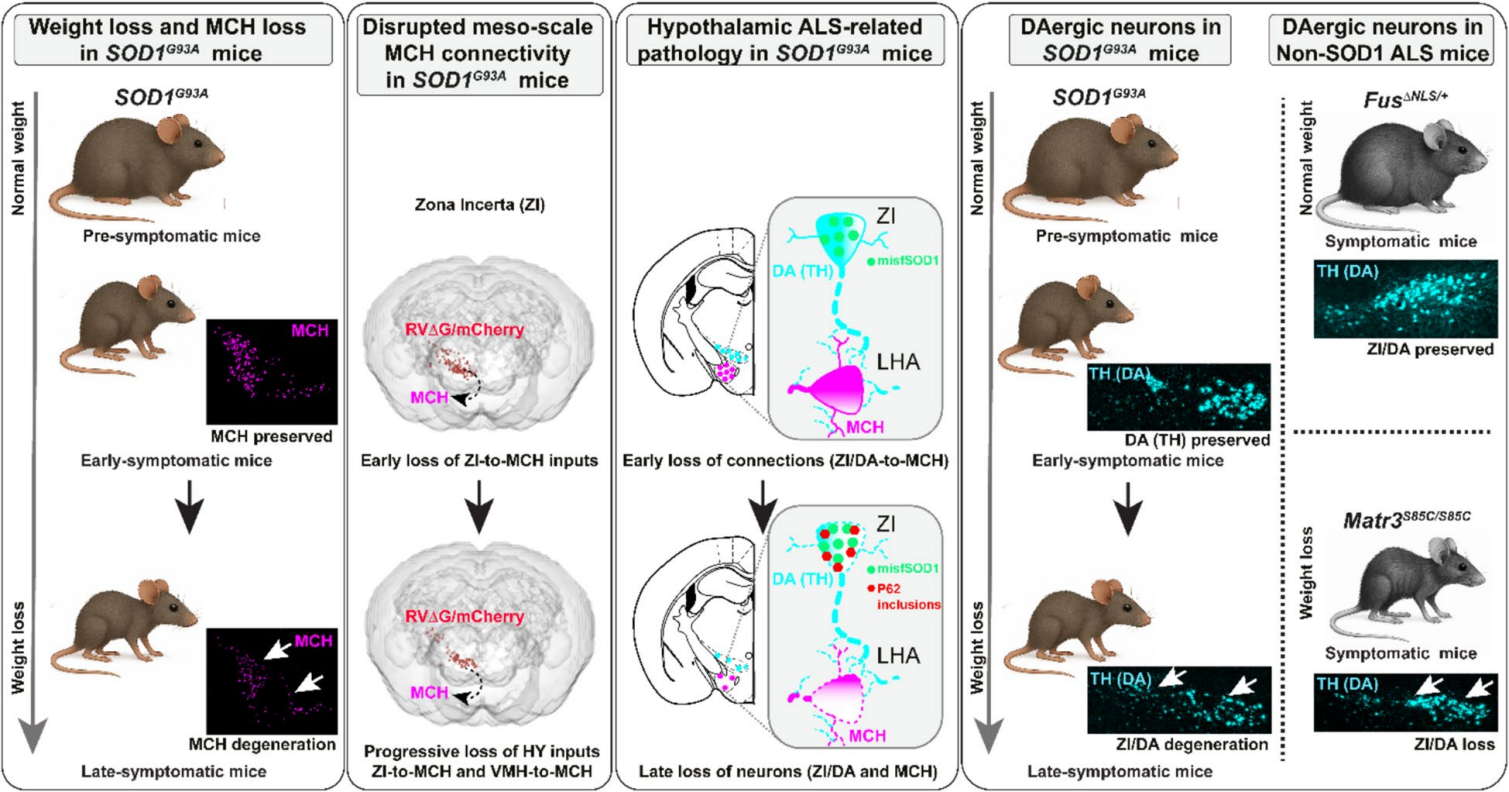
Early loss of ZI–MCH connectivity is associated with the subsequent vulnerability of hypothalamic neurons and metabolic imbalance in ALS. The schematic summary model illustrates the spatiotemporal cascade of hypothalamic network disruption in ALS. In *SOD1*^*G93A*^ mice, body weight reduction begins in the early-symptomatic stage, while MCH neurons in the LHA degenerate only in the late disease stage, coinciding with prominent weight loss (first panel). Whole-brain monosynaptic rabies tracing reveals an early and selective reduction of ZI inputs to MCH neurons that precedes MCH degeneration (second panel). Histopathological analyses show early ALS-related pathology in the ZI, including the accumulation misfolded SOD1, followed by the degeneration of ZI/DA and MCH neurons, together with the appearance of p62/SQSTM1 inclusions in the later disease stage (third panel). DAergic neuron vulnerability differs across ALS models in parallel with their body-weight phenotypes (fourth panel)

In this study, we provide converging evidences from ALS murine models identifying a selectively affected hypothalamic subnetwork associated with ALS-related MCH vulnerability and metabolic dysregulation, in particular an early disruption of DAergic and GABAergic connections from the ZI to the MCH neurons (Fig. 11). These findings advance our understanding of ALS pathogenesis and open new opportunities for pharmacological interventions to mitigate weight loss in the disease.

Metabolic dysfunction, including weight loss and hypermetabolism [29, 36, 50, 55, 72], precedes the onset of ALS and is associated with faster disease progression and shorter survival. While the hypothalamus has emerged as a key brain region implicated in ALS-related metabolic disturbances [1, 28], the cellular and network mechanisms driving this vulnerability remain largely unexplored.

### A broad vulnerability of hypothalamus in ALS

First, we provide novel insights into the full scope of vulnerability of hypothalamic peptidergic neurons in the *SOD1*^*G93A*^ ALS mouse model. Our results show that anatomically and functionally distinct neurons expressing MCH (Fig. 11, first panel), Galanin, CART, and POMC are significantly lost in the late stage of disease, after the onset of weight loss. While MCH degeneration, associated with negative energy balance due to reduced food intake, has been reported previously in ALS [7], the degeneration of other subpopulations has not yet been described. Nevertheless, these populations are integrated into common energy-regulating circuits and fulfil distinct roles [3, 35, 80]. LHA-Galanin neurons increase food-seeking behaviour [66] while ARH-POMC neurons mediate appetite suppression via the melanocortin pathway [76]. Interestingly, CART expression co-localizes with MCH (LHA) and POMC (ARH) neurons and induces dual, location-dependent anabolic and catabolic effects, respectively [43]. The multiple neuropeptidergic degeneration in our study echoes the observation of Gabery and collaborators [25] and of Bergh et al. [6], who showed a concomitant loss of LHA orexin-producing neurons, and of PVN oxytocin neurons in ALS patients and mice, which correlated with changes in eating behaviour. Importantly, these findings align with limited responsiveness to drugs targeting the individual hypothalamic populations either by MCH supplementation [7, 31] or through the POMC/AgRP pathway [22]. Our findings expand on previous reports of hypothalamic vulnerability in ALS, and reveal a broader range of affected neuronal subpopulations. Together, the early weight loss and the late-onset, widespread hypothalamic degeneration suggest that the metabolic phenotype in ALS is not solely due to the loss of individual neuron types, but rather reflects an early, network-wide dysfunction.

### An early vulnerability of ZI-to-MCH network in ALS

Next, we examined the integrity of MCH upstream network in *SOD1*^*G93A*^ mice along two disease stages using retrograde modified-rabies tracing to map brain-wide monosynaptic inputs to MCH neurons (being the most affected population). Our tracing study revealed that global MCH connectivity remained intact at the early-symptomatic stage of disease, despite the presence of toxic SOD1, with the hypothalamus (HY) as the primary input source, alongside contributions from deep cerebral nuclei (CNU), cortex (CTX), and midbrain (MB). Notably, the dose-dependent effects of rabies tracing confirmed the robustness of these projections, as higher viral loads resulted in an increased representation of weaker or more distal connections in recent reports [37, 74]. Importantly, this MCH connectivity pattern matched the tracing study in *WT* mice by Gonzalez et al. [26]. In contrast, at the late-symptomatic stage of disease, correlated with MCH degeneration, the large-scale architecture of MCH network was affected by mutant SOD1 expression leading to reduction in HY inputs to MCH neurons and to increase in inputs from CNU subregions.

Despite that early in disease large-scale connectivity was stable, analysis at meso-scale resolution revealed a significant reduction of afferent connections from zona incerta (ZI) to MCH neurons (Fig. 11, second panel). Notably, this finding was replicated in a mice cohort injected with a higher rabies viral load. As disease advanced the loss of ZI inputs persisted and was accompanied by a decline of projections originating from ventromedial hypothalamus (VMH), indicating a progression of circuit disruptions. Together, these results identify the ZI-to-MCH network as an early site of selective vulnerability that precedes MCH degeneration.

Of note, ALS drives an early and progressive redistribution of CNU connectivity, starting with increased lateral septum (LS) inputs and expanding over time to substantia innominata (SI) projections. This echoes the most recent magnetic resonance imaging (MRI) study in ALS patients showing increased hypothalamic functional connectivity with caudate nuclei correlated with disease severity [23]. Secondary motor cortex (MOs) disconnections are revealed under high rabies titers and may reflect early cortical circuit vulnerability reported in this and other ALS models [5, 12, 17].

### What could be the relevance of ZI-to-MCH connections in metabolism?

Although our study does not directly test the functional consequences of ZI-to-MCH disconnection, existing literature allows us to speculate on its potential metabolic relevance. The ZI is a largely inhibitory subthalamic region conserved among mammals [65] composed of neurochemically mixed neurons [54, 57] and with extensive connections (often reciprocal) that cover approximately the entire brain (for a review, see [4]) allowing the ZI to modulate the function of numerous neural networks.

In our study, we found that ZI neurons projecting to MCH neurons include both GABAergic and DAergic populations, and that DAergic neurons degenerate following their disconnections from MCH neurons. Both vulnerable populations, MCH and ZI/DAergic, received direct projections from ALS-affected layer 5 cortical neurons. Indeed, the ZI is predominantly GABAergic in mice and other taxa [45, 83], but a subset of ZI/GABAergic neurons co-express the enzyme TH and produce DA, restricted to the rostral ZIr near the midline and lacking any other neuropeptides [57]. In line with our tracing results, Yang et al., recently showed that in mice ZI/GABAergic neurons have brain-wide bidirectional projections, whereas the rostral ZIr and medial ZIm have strong interconnections with the LHA, and also receive projections from layer 5 motor cortex neurons [83]. In 2025, Bono et al., published the whole‑brain output connectome of mouse ZI/DAergic and confirmed that ZI/DAergic projections closely mirror those of ZI/GABAergic neurons, with robust outputs to the LHA, consistent with our data [8]. Pivotal neuroanatomical studies in rodents have reported the existence of bidirectional projections between the ZI and both the LHA and VMH hypothalamic areas, as well as prominent cortico-incertal projections originating from the layer 5 neurons (for a review, see [4]). Similar connectivity patterns were observed in primates by Haber et al.: connections of ZIr and the dorsolateral prefrontal cortex [34], which was confirmed in humans by Saluja et al. using high-resolution diffusion MRI, showing that ZIr receives projections from prefrontal areas, while the caudal ZI is primarily connected to motor regions [67].

To the best of our knowledge we are the first to report the connections between ZI GABAergic and DAergic neurons and a specific population in LHA, MCH neurons, as well as to show that these inputs are primarily affected at the synaptic level by disease and prior to MCH loss in *SOD1*^*G93A*^ mice. The early loss of connectivity was accompanied by the accumulation of ALS-related pathological markers, and by a neuroinflammatory response in ZI, while degeneration of DAergic neurons occurred later in disease progression. A similar pathology in ZI of *Matr3*^*S85C/S85C*^ mice and its absence in *Fus*^*∆NLS/*+^ mice were consistent with their respective body-weight phenotypes (Fig. 11, fourth panel). Altogether, supported the hypothesis that not only the MCH dysfunction but the metabolic phenotype in ALS is closely associated with the early breakdown of specific hypothalamic networks, rather than arising solely from the degeneration of individual neuronal populations.

In fact, emerging functional research has recognized ZI as an integrative node for global modulation of behaviours and physiological states (especially feeding), depending on its specific input–output connectivity patterns (for a review, see [79]). Early studies indicated that lesions of the rostral ZI led to decreased food intake [20]. More specifically, ZI/GABAergic signalling has been implicated in feeding [88], hunting [89], and in regulation of the sleep–wake cycle [47]. While acute optogenetic stimulation of projections from ZI/GABAergic neurons to the anorexigenic paraventricular thalamus (PVT) evoked binge-like eating, particularly of high-fat food, the chronic stimulation induced persistent overeating and weight gain [88]. In contrast, the ablation of ZI/GABAergic neurons resulted in long-term reductions in both food intake and body weight gain [88]. Complementing this unexpectedly robust orexigenic potential of the ZI/GABAergic neurons, chemogenetic activation of ZI/DAergic neurons was shown to enhance the motivation for feeding by increasing meal frequency without affecting the overall food intake, mainly through their projections to the PVT [84]. Conversely, mice with global DA deficiency were reported to be aphagic with severe starvation [90].

Given the dense reciprocal connectivity between the ZI and MCH neurons shown by our tracing study, it is plausible that ZI inputs may modulate MCH neuronal activity in a manner akin to its influence on PVT neurons—i.e., cell-type-specific regulation of firing dynamics through both fast-GABAergic inhibition and slower DAergic neuromodulation. However, the published data are controversial, Conductier et al., showed that MCH neurons receive mainly GABAergic inputs and that DA modulates these inputs in a complex manner: at low concentrations, DA activates D1-like receptors, promoting presynaptic GABA activity, whereas, at higher concentrations, D2-like receptor activation inhibits presynaptic GABA activity, but overall results in down-regulation of MCH release [18]. In contrast, injections of D1 and D2 receptor agonists in LHA did not affect MCH mRNA levels [85]. Further exploration of ZI-to-MCH functional connectivity could clarify the broader role of ZI in orchestrating hypothalamic control of energy homeostasis.

### Pathological mechanisms underlying ZI-to-MCH disconnection

At the cellular level, an early accumulation of a toxic misfolded SOD1 protein (at P45 in *SOD1*^*G93A*^ mice) and P62/SQSTM1 inclusions, a marker of defective autophagy, was selectively enhanced within the ZI, and in DAergic neurons (Fig. 11, third panel). While ALS-related pathology was present pre-symptomatically and to the similar extent in the ZI as in the motor cortex pointing to shared disease mechanisms, other nearby regions were almost completely speared. Given that protein aggregation and impaired clearance mechanisms [78] are hallmark features of ALS and contribute to motor neuron vulnerability, our findings indicate that a similar mechanism may drive neuronal disconnection, predating the degeneration. Based on our data, it is not possible to distinguish whether the ALS pathology is intrinsic to ZI or reflects a spread from motor regions to connected hypothalamic areas, as proposed by Brettschneider J et al. [11]. The latter is further corroborated by our anterograde tracing data showing direct targeting of ZI and DAergic neurons by motor cortical projections, with a notably higher density of inputs compared to LHA and MCH neurons.

In addition, we observed pronounced astrogliosis specifically in the ZI, which preceded both, ZI disconnection from MCH neurons and neuronal degeneration, in contrast to the absence of microglial activation in this area even at the advanced disease stages in *SOD1*^*G93A*^ mice. Together with our findings of preserved ZI/DAergic neurons and absent astrogliosis in *Fus*^*∆NLS/*+^ mice, the results support a similar astrocyte-mediated non-cell-autonomous toxicity for ZI neurons as shown for ALS motor neurons [73]. As astrocytes are well known to play roles in neurotransmitter homeostasis and in metabolic support, our findings suggest that specific glial cells may contribute to the early disintegration of the ZI-MCH circuit in ALS.

Importantly, while our data establish a clear temporal hierarchy of hypothalamic pathology and connectivity loss, they do not directly demonstrate that ZI disconnection causes degeneration of hypothalamic neurons, and future functional or interventional studies will be required to address causality. One of the key limitations of this study is the absence of direct human data validating the observed DAergic degeneration in the ZI in ALS cases. However, a DA deficit in the nigrostriatal system has been reported in sporadic ALS patients presenting with coexisting idiopathic parkinsonism [61] or with clinical forms of Parkinson's disease (PD) [56]. Interestingly, deep brain stimulation (DBS) of the ZI alleviates motor symptoms in PD [15, 59], but also affects food intake in patients as binge-like eating and weight gain have been reported as frequent side effects and could be beneficial for ALS patients [87]. Our work gains further relevance from recent clinical trials showing benefits of rasagiline (a DA degradation inhibitor) for patients with ALS [70]. While our study in ALS mouse models suggests that DAergic neurons play an early role in metabolic dysregulation, future studies incorporating patient data are essential to establish their relevance to human ALS pathology and strengthen the translational significance of our findings.

## Conclusions

Our study identifies an early and selective breakdown of the ZI–MCH hypothalamic network as a potential mechanism underlying MCH vulnerability and the resulting metabolic imbalance. Our tracing, histological, and cross-model analyses show that DAergic and GABAergic inputs from the ZI to MCH neurons are lost first, coinciding with the early occurrence of ALS-related pathology in the ZI, and are followed by the later degeneration of both MCH and ZI/DAergic neurons. These results therefore establish a spatiotemporal cascade of metabolic network collapse, and support a model in which ZI pathology most likely disrupts DA/GABAergic modulation of MCH neurons, and downstream hypothalamic circuits ultimately fail to maintain energy homeostasis. By uncovering this hierarchical network collapse, our study shifts the view of ALS-associated metabolic imbalance from the degeneration of hypothalamic neurons to a circuit-level mechanism and highlights ZI–MCH signalling as a promising therapeutic target to counteract weight loss and metabolic decline in ALS.

## Supplementary Information

Supplementary Material 1.

## Data Availability

All data supporting this study’s findings are available either within the paper or from the corresponding author on reasonable request.

## Abbreviations

AAV: Adeno-associated virus
ACA: Anterior cingulate area
ACB: Nucleus accumbens
AgRP: Agouti-related peptide
ALS: Amyotrophic lateral sclerosis
ARH: Arcuate nucleus
BMI: Body mass index
BST: Bed nucleus of the stria terminalis
CART: Cocaine- and amphetamine-regulated transcript
CNU: Deep cerebral nuclei
CTX: Cortex
DA: Dopamine
DAT: Dopamine transporter
DBH: Dopamine β-hydroxylase
DBS: Deep brain stimulation
DMH: Dorsomedial nucleus of hypothalamus
DOPA: Decarboxylase
GABA: Gamma-aminobutyric acid
GAL: Galanin
HCRT: Orexin/hypocretin
HT: High titer
HY: Hypothalamus
ILA: Infralimbic area
ISH: *in situ* Hybridization
LHA: Lateral hypothalamic area
LMN: Lower motor neurons
LS: Lateral septum
LT: Low titer
L5 PN: Cortical layer 5 long projection neurons
MB: Midbrain
MCH: Melanin-concentrating hormone
misfSOD1: Misfolded SOD1
MO: Motor cortex
MOs: Secondary motor cortex (MOs)
MRN: Midbrain reticular nucleus
NpY: Neuropeptide Y
ORB: Orbital area
PAG: Periaqueductal gray
PD: Parkinson's disease
POMC: Pro-opiomelanocortin
PVT: Paraventricular thalamus
ROI: Region of interest
SCm: Motor related superior colliculus
SI: Substantia innominata
SNr: Substantia nigra
SST: Somatostatin
SUBv: Ventral subiculum
Th: Tyrosine hydroxylase
TH: Thalamus
TU: Tuberal nucleus
UMN: Upper motor neurons
VGAT: Vesicular GABA transporter
VGlut2: Vesicular glutamate transporter
VMH: Ventromedial hypothalamic nucleus
VTA: Ventral tegmental area
ZI: Zona incerta
