# Supplementary material for: Large-scale mapping of the MCH network in ALS mice reveals the vulnerability of dopaminergic and GABAergic neurons in zona incerta
Source: Acta Neuropathol Commun. 2026 Feb 6;14:46. doi: 10.1186/s40478-026-02231-z (PMC12930930; doi:10.1186/s40478-026-02231-z)
Supplement: Supplementary file 1 — Supplementary Material 1. [file 40478_2026_2231_MOESM1_ESM.pdf]

Supplemental Materials for

Large-scale mapping of the MCH network in ALS mice reveals the vulnerability of dopaminergic and GABAergic neurons in Zona Incerta

Jelena Scekic-Zahirovic *et al.*

*Co-corresponding authors: Francesco Roselli, francesco.roselli@uni-ulm.de
Jelena Scekic-Zahirovic, jelena.scekic-zahirovic@dzne.de

This PDF file includes:

Figures S1 to S12
Tables S1 to S3

Figure S1

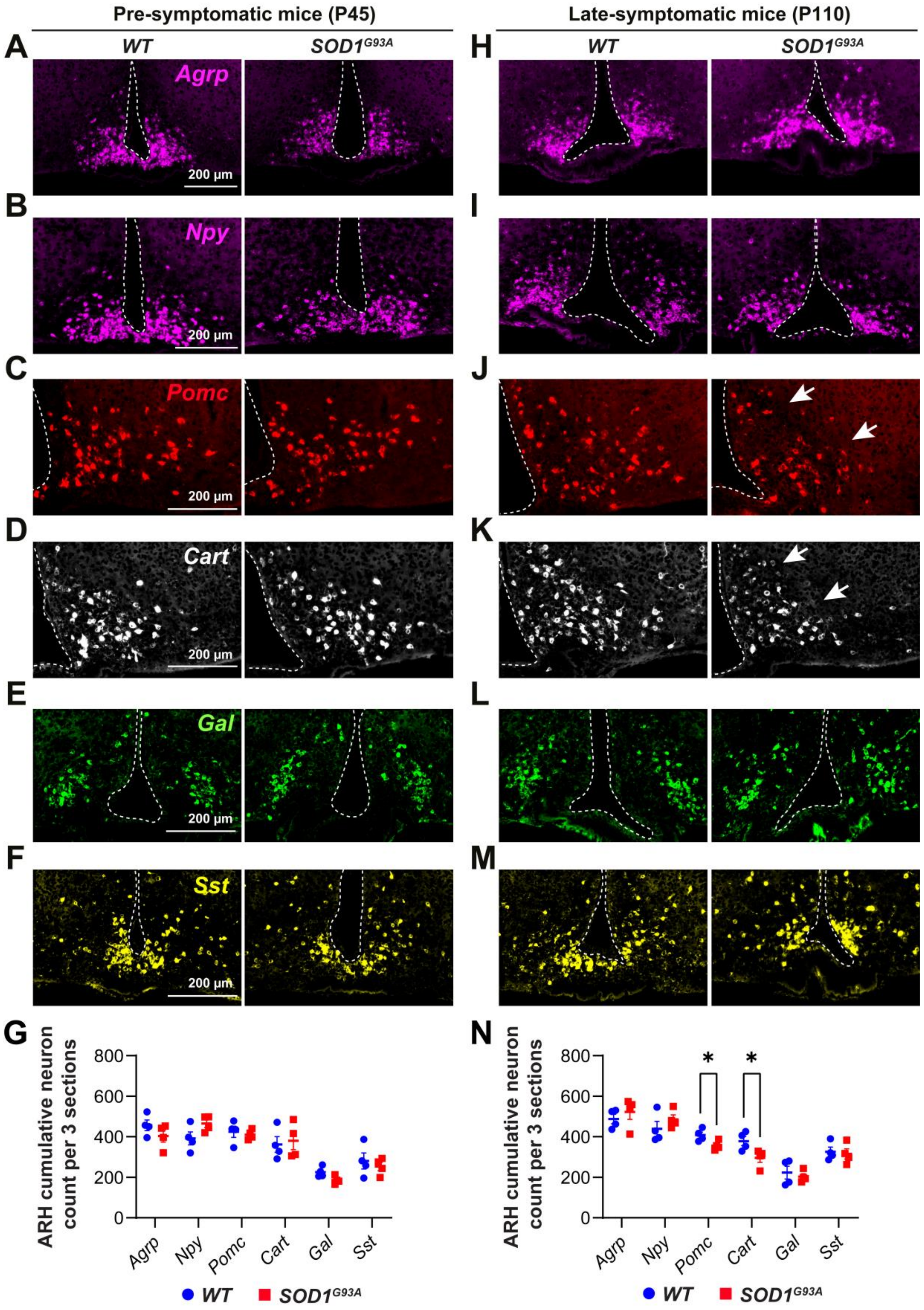

Pre-symptomatic mice (P45)
Late-symptomatic mice (P110)
A
B
C
D
E
F
G
H
I
J
K
L
M
N
WT
SOD1G93A
Agrp
Npy
Pomc
Cart
Gal
Sst
200 µm
ARH cumulative neuron count per 3 sections
800
600
400
200
0
*
*
Agrp
Npy
Pomc
Cart
Gal
Sst
WT
SOD1G93A

Fig. S1. Loss of POMC and CART populations in the ARH in late-symptomatic *SOD1^{G93A}* mice.

A-F, H-M. Representative images of the ARH of P45 (**A-F**), and P110 (**H-M**) *WT* (left panels) and *SOD1^{G93A}* (right panels) mice showing neuronal mRNA expression of specific neuropeptides: **A, H**. *Agrp* for agouti-related protein (in magenta), **B, I**. *Npy* for neuropeptide Y (in magenta), **C**. *Pomc* for pro-opiomelanocortin (in red), **D, K**. *Cart* (in grey), **E, L.** *Gal* (in green) and **F, M**. *Sst* for somatostatin (in yellow).

G, N. Quantification of neuropeptidergic populations located in the ARH of P45 (**G**), and P110 (**N**) *SOD1^{G93A}* (red) and *WT* (blue) mice. *Pomc* (p=0.03) and *Cart* (p=0.04) expressing neurons are significantly reduced in *SOD1^{G93A}* mice at P110 – late-symptomatic disease stage.

N=4 animals per genotype. (*) p-value<0.05, Multiple unpaired t-test per neuronal population. Data are displayed as the mean ± SEM, with each data point representing a single animal. Scale bars: 200 μm.

Figure S2

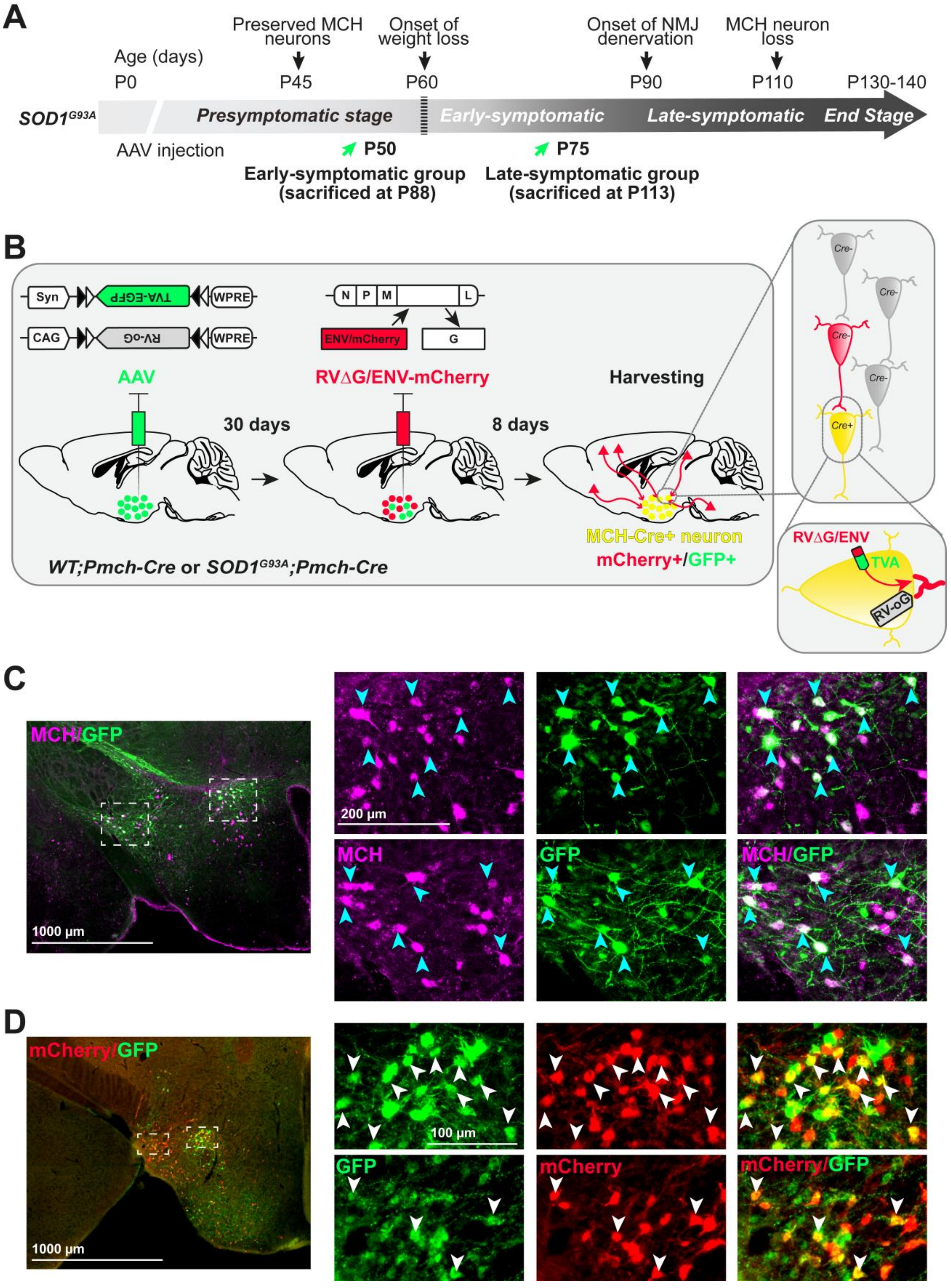
A
Preserved MCH neurons
Onset of weight loss
Onset of NMJ denervation
MCH neuron loss
Age (days)
P0
P45
P60
P90
P110
P130-140
SOD1G93A
Presymptomatic stage
Early-symptomatic
Late-symptomatic
End Stage
AAV injection
P50
Early-symptomatic group (sacrificed at P88)
P75
Late-symptomatic group (sacrificed at P113)
B
Syn
TVA-EGFP
WPRE
CAG
RV-oG
N P M L
ENV/mCherry
G
AAV
RVΔG/ENV-mCherry
Harvesting
30 days
8 days
MCH-Cre+ neuron
mCherry+/GFP+
WT;Pmch-Cre or SOD1G93A;Pmch-Cre
Cre-
Cre+
RVΔG/ENV
TVA
RV-oG
C
MCH/GFP
1000 µm
200 µm
MCH
GFP
MCH/GFP
D
mCherry/GFP
1000 µm
100 µm
GFP
mCherry
mCherry/GFP

Fig. S2. Experimental strategy for the selective retrograde tracing of whole-brain monosynaptic inputs to MCH neurons.

A. Schematic of the disease stages in *SOD1*^{G93A};*Pmch*-Cre mice.

B. The scheme outlines the experimental design: AAV constructs expressing Cre-dependent TVA-EGFP and rabies oG protein (helper viruses) were injected at P50 (early-symptomatic mice) or P75 (late-symptomatic mice), followed by an injection of pseudotyped and modified rabies virus (RVΔG/ENV-mCherry) after 30 days and the mice being harvested 8 days later. The enlarged insets illustrate the mechanism of triple viral infection of Cre+ MCH neurons (starter neurons – yellow) and their monosynaptic inputs (red). The modified rabies virus can only cross synapses when it is completed by the oG protein inside the Cre+ MCH neuron.

C. Representative images of a coronal hypothalamic section (overview) and enlarged insets (white dashed line rectangle) from a *Pmch-Cre* mouse depict the selectively infected hypothalamic MCH neurons (magenta) co-expressing EGFP (green) delivered by AAV vectors, as demonstrated in **B** (cyan arrowheads).

D. An example of a hypothalamic section and enlarged insets showing MCH neurons (starter neurons – yellow), double positive for EGFP and mCherry (white arrowheads), and their direct, monosynaptic input neurons expressing only the mCherry reporter (red).

Scale bars: 1000 μm (for lower magnification images) and 100 μm (for higher magnification images).

Figure S3

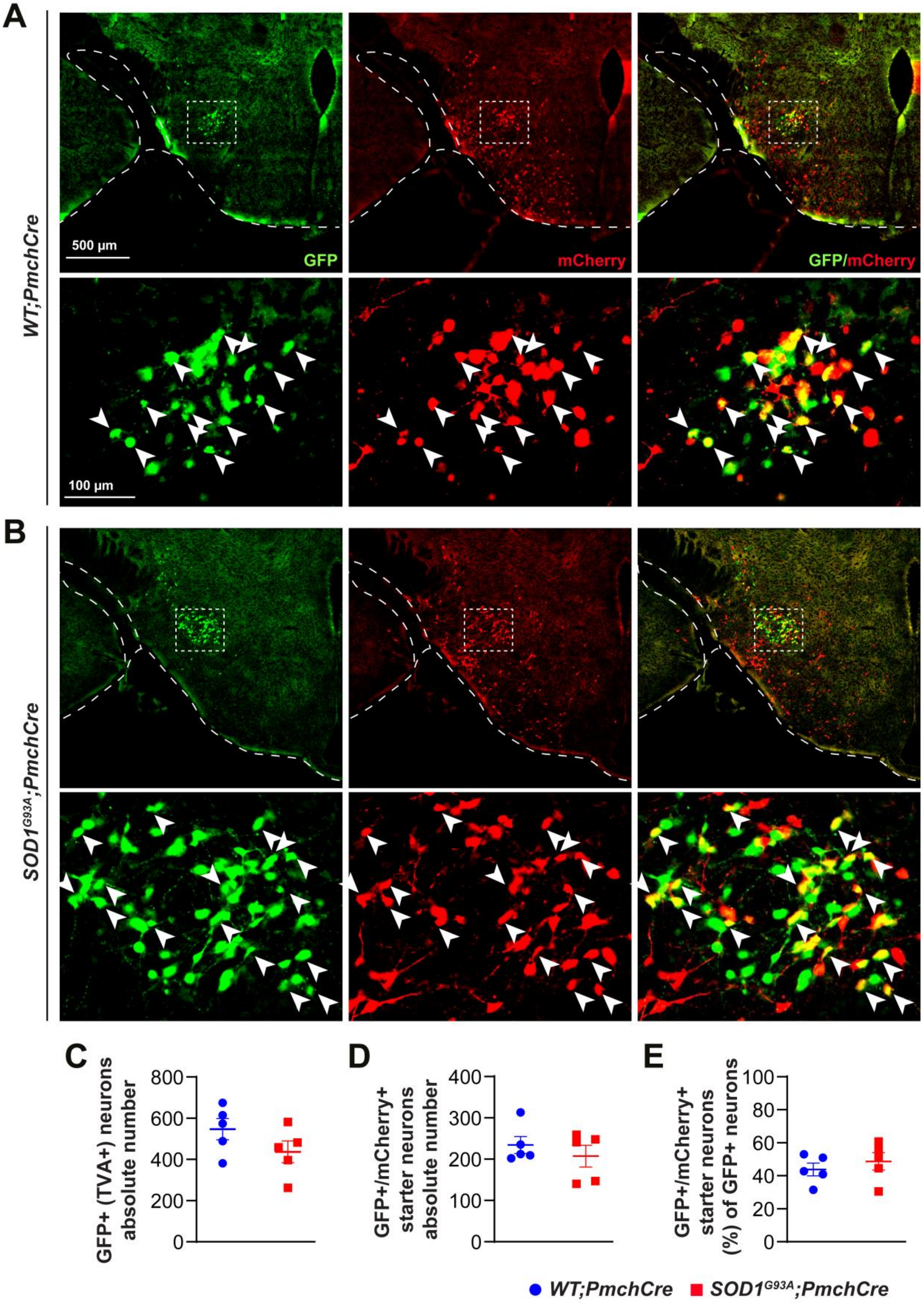
A
WT;PmchCre
500 µm
GFP
mCherry
GFP/mCherry
100 µm
B
SOD1G93A;PmchCre
C
GFP+ (TVA+) neurons absolute number
800
600
400
200
0
D
GFP+/mCherry+ starter neurons absolute number
400
300
200
100
0
E
GFP+/mCherry+ starter neurons (%) of GFP+ neurons
100
80
60
40
20
0
WT;PmchCre
SOD1G93A;PmchCre

Fig. S3. Starter neurons engineered to enable the initial rabies infection and one-step viral spread to presynaptic partners.

A-B. Representative images of a coronal section of the hypothalamus (overview) and of the LHA (white dashed line square), depicting the starter neurons (yellow, white arrowheads) double positive for TVA-EGFP (green) and the mCherry-tagged modified rabies virus (red) in late-symptomatic *WT;Pmch*-Cre (**A**) and *SOD1*^{G93A}*;Pmch*-Cre (**B**) mice.

C-E. The quantification plots show: a comparable number of neurons infected with TVA-EGFP (**C**), an unchanged number of neurons co-infected (starter neurons) with TVA-EGFP and modified rabies (**D**) and the same percentage of starter neurons (**E**) between genotypes.

N=5 animals per genotype. Two-tailed unpaired Student's t-test. Data are displayed as the mean ± SEM, with each data point representing a single animal. Scale bars: 500 µm (for lower magnification images), 100 µm (for higher magnification images).

Figure S4

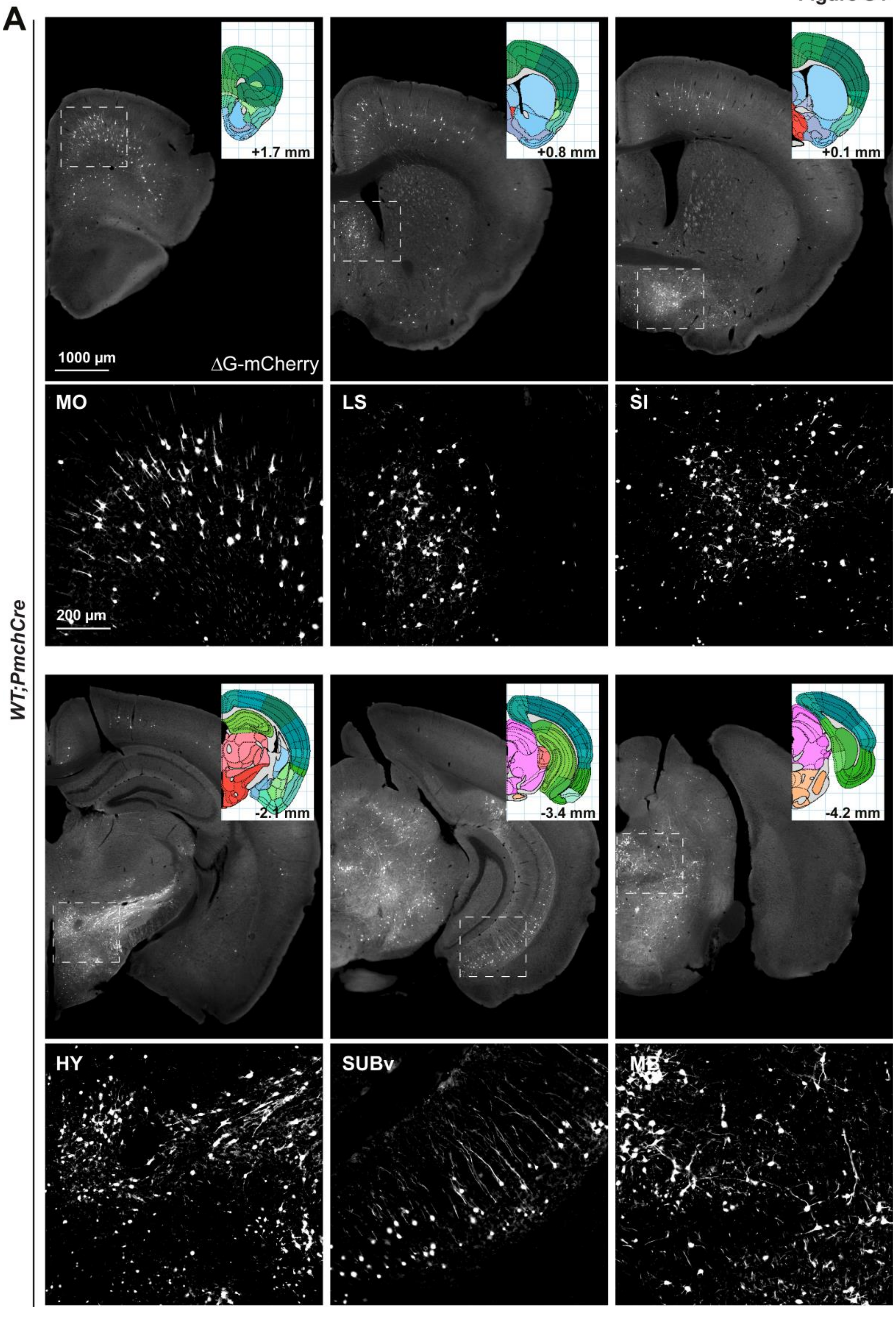
A
WT;PmchCre
+1.7 mm
+0.8 mm
+0.1 mm
1000 µm
ΔG-mCherry
MO
LS
SI
200 µm
-2.1 mm
-3.4 mm
-4.2 mm
HY
SUBv
MB

Fig. S4. Distribution of monosynaptic rabies-traced inputs to MCH neurons throughout the whole brain.

A. Representative native fluorescence images of coronal brain sections showing the distribution of mCherry-labelled input neurons to MCH neurons throughout the whole brain of an 88-day-old *WT;Pmch-Cre* mouse. Following modified rabies injection into the LHA, labelled neurons were consistently observed from Bregma +3 mm to Bregma −5 mm. The enlarged insets represent input neurons from the motor cortex (MO), lateral septum (LS), substantia innominata (SI), hypothalamus (HY), ventral subiculum (SUBv) and midbrain (MB). The slice schematics represent images from the Openbrainmap website corresponding to the Bregma positions of coronal brain sections along the anterior-posterior axis.

Scale bar: 1000 μm (for lower magnification images) and 200 μm (for higher magnification images).

Figure S5

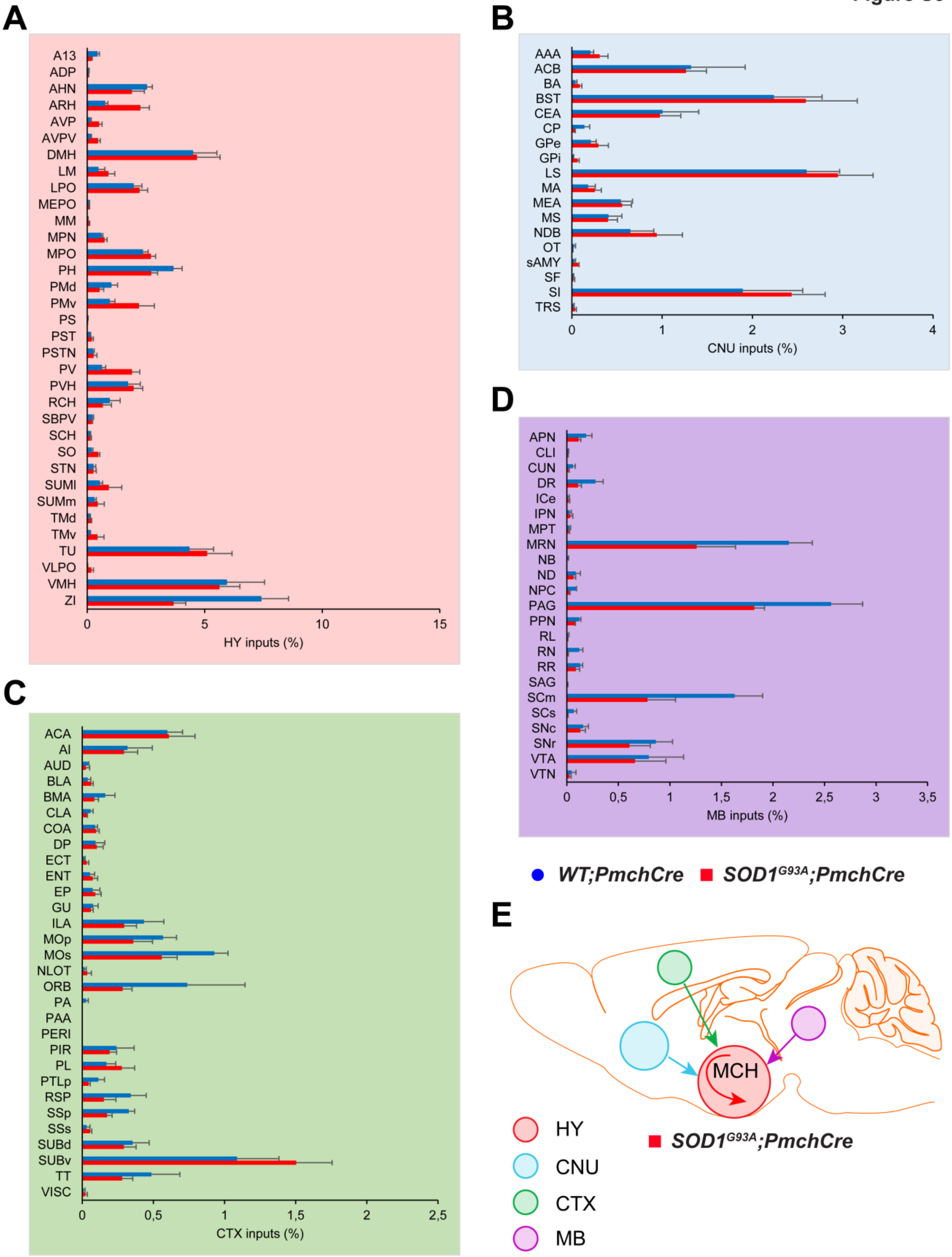
A
A13
ADP
AHN
ARH
AVP
AVPV
DMH
LM
LPO
MEPO
MM
MPN
MPO
PH
PMd
PMv
PS
PST
PSTN
PV
PVH
RCH
SBPV
SCH
SO
STN
SUMl
SUMm
TMd
TMv
TU
VLPO
VMH
ZI
0
5
10
15
HY inputs (%)
B
AAA
ACB
BA
BST
CEA
CP
GPe
GPi
LS
MA
MEA
MS
NDB
OT
sAMY
SF
SI
TRS
0
1
2
3
4
CNU inputs (%)
C
ACA
AI
AUD
BLA
BMA
CLA
COA
DP
ECT
ENT
EP
GU
ILA
MOp
MOs
NLOT
ORB
PA
PAA
PERI
PIR
PL
PTLp
RSP
SSp
SSs
SUBd
SUBv
TT
VISC
0
0,5
1
1,5
2
2,5
CTX inputs (%)
D
APN
CLI
CUN
DR
ICe
IPN
MPT
MRN
NB
ND
NPC
PAG
PPN
RL
RN
RR
SAG
SCm
SCs
SNc
SNr
VTA
VTN
0
0,5
1
1,5
2
2,5
3
3,5
MB inputs (%)
WT;PmchCre
SOD1G93A;PmchCre
E
MCH
HY
CNU
CTX
MB
SOD1G93A;PmchCre

Fig. S5. Meso-scale whole-brain mapping of neuronal inputs to MCH neurons traced using a low titer of ΔG rabies virus in early-symptomatic mice.

A-D. High-resolution quantification of monosynaptic inputs to MCH neurons across 130 brain areas of the hypothalamus (**A**), cerebral nuclei (**B**), cortex (**C**) and midbrain (**D**). Data are represented as the mean ± SEM of the proportion of inputs from each area/region in early-symptomatic *WT*;*Pmch*-Cre and *SOD1*^{G93A};*Pmch*-Cre mice (N=4 per genotype) injected with a low titer of modified rabies virus (LT). **e**. Schematic showing the relative contributions of four large-scale areas inputs to MCH neurons. The size of each circle represents the proportion of total whole-brain inputs and the arrows indicate the unchanged large-scale inputs in *SOD1*^{G93A};*Pmch*-Cre mice (thin line). Anatomical abbreviations are based on the Allen Brain Atlas (ABA), and are fully defined in **Table S3**.

Figure S6

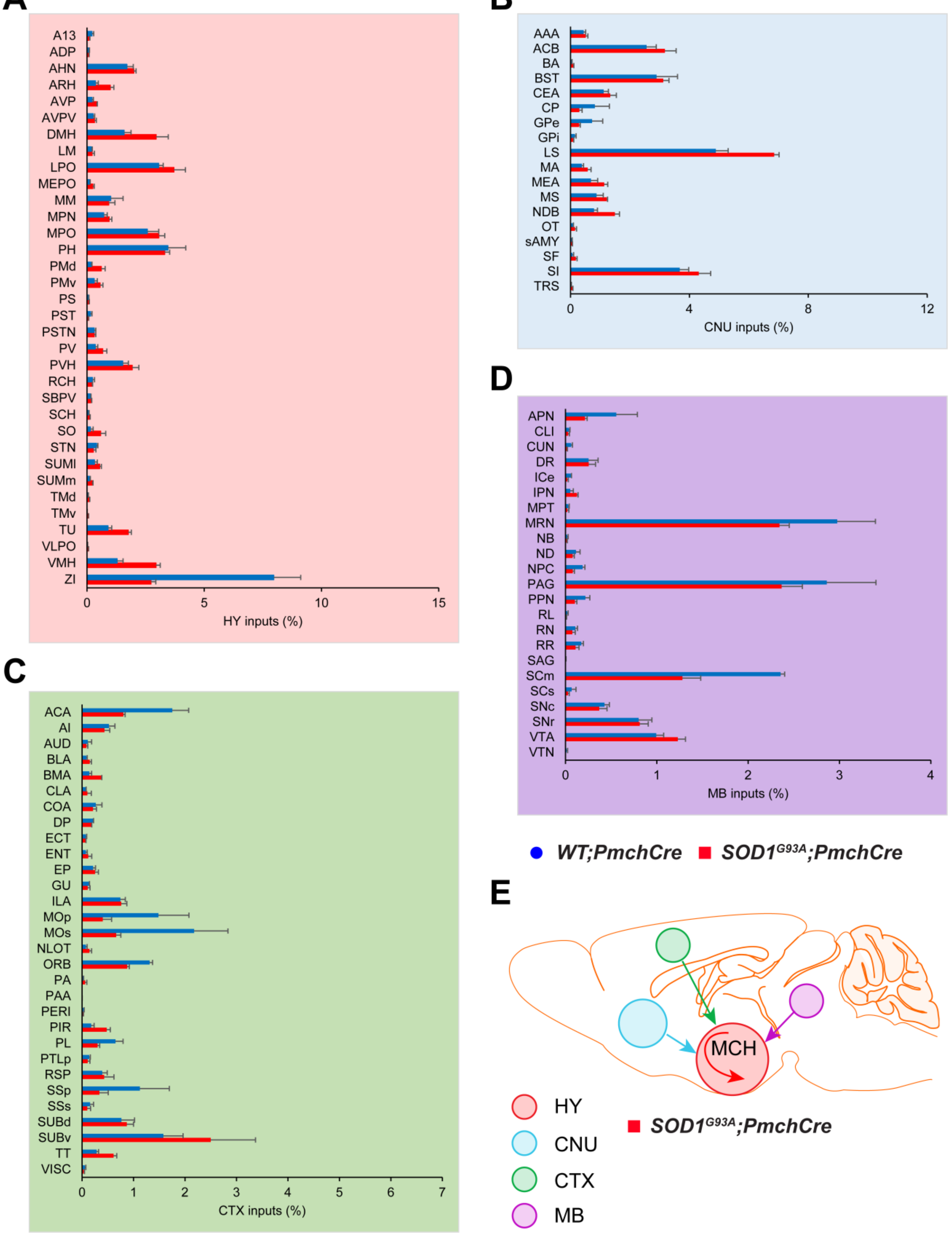
A
A13
ADP
AHN
ARH
AVP
AVPV
DMH
LM
LPO
MEPO
MM
MPN
MPO
PH
PMd
PMv
PS
PST
PSTN
PV
PVH
RCH
SBPV
SCH
SO
STN
SUMl
SUMm
TMd
TMv
TU
VLPO
VMH
ZI
0
5
10
15
HY inputs (%)
B
AAA
ACB
BA
BST
CEA
CP
GPe
GPi
LS
MA
MEA
MS
NDB
OT
sAMY
SF
SI
TRS
0
4
8
12
CNU inputs (%)
C
ACA
AI
AUD
BLA
BMA
CLA
COA
DP
ECT
ENT
EP
GU
ILA
MOp
MOs
NLOT
ORB
PA
PAA
PERI
PIR
PL
PTLp
RSP
SSp
SSs
SUBd
SUBv
TT
VISC
0
1
2
3
4
5
6
7
CTX inputs (%)
D
APN
CLI
CUN
DR
ICe
IPN
MPT
MRN
NB
ND
NPC
PAG
PPN
RL
RN
RR
SAG
SCm
SCs
SNc
SNr
VTA
VTN
0
1
2
3
4
MB inputs (%)
WT;PmchCre
SOD1G93A;PmchCre
E
MCH
HY
CNU
CTX
MB
SOD1G93A;PmchCre

Fig. S6. Meso-scale whole-brain mapping of neuronal inputs to MCH neurons traced using a high titer of ΔG rabies virus in early-symptomatic mice.

A-D. High-resolution quantification of monosynaptic inputs to MCH neurons across 130 brain areas of the hypothalamus (**A**), cerebral nuclei (**B**), cortex (**C**) and midbrain (**D**). Data are represented as the mean ± SEM of the proportion of inputs from each area/region in early-symptomatic *WT*;*Pmch*-Cre and *SOD1*^{G93A};*Pmch*-Cre mice (N=3 per genotype) injected with a high titer of modified rabies virus (HT). **e**. Schematic showing the relative contributions of four large-scale areas inputs to MCH neurons. The size of each circle represents the proportion of total whole-brain inputs and the arrows indicate the unchanged large-scale inputs in *SOD1*^{G93A};*Pmch*-Cre mice (thin line). Anatomical abbreviations are based on the Allen Brain Atlas (ABA), and are fully defined in **Table S3**.

Figure S7

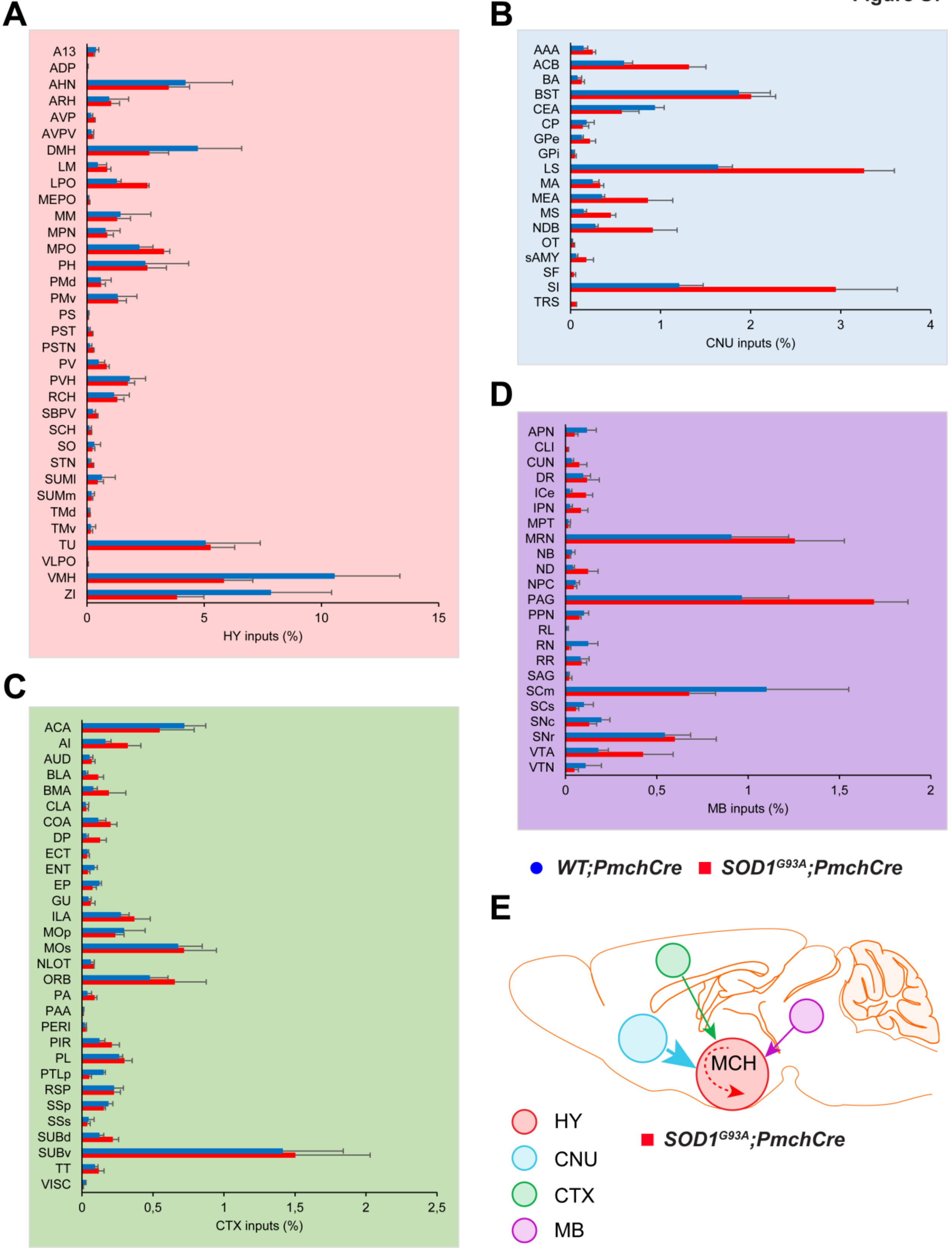
A
A13
ADP
AHN
ARH
AVP
AVPV
DMH
LM
LPO
MEPO
MM
MPN
MPO
PH
PMd
PMv
PS
PST
PSTN
PV
PVH
RCH
SBPV
SCH
SO
STN
SUMl
SUMm
TMd
TMv
TU
VLPO
VMH
ZI
0
5
10
15
HY inputs (%)
B
AAA
ACB
BA
BST
CEA
CP
GPe
GPi
LS
MA
MEA
MS
NDB
OT
sAMY
SF
SI
TRS
0
1
2
3
4
CNU inputs (%)
C
ACA
AI
AUD
BLA
BMA
CLA
COA
DP
ECT
ENT
EP
GU
ILA
MOp
MOs
NLOT
ORB
PA
PAA
PERI
PIR
PL
PTLp
RSP
SSp
SSs
SUBd
SUBv
TT
VISC
0
0,5
1
1,5
2
2,5
CTX inputs (%)
D
APN
CLI
CUN
DR
ICe
IPN
MPT
MRN
NB
ND
NPC
PAG
PPN
RL
RN
RR
SAG
SCm
SCs
SNc
SNr
VTA
VTN
0
0,5
1
1,5
2
MB inputs (%)
WT;PmchCre
SOD1G93A;PmchCre
E
MCH
HY
CNU
CTX
MB
SOD1G93A;PmchCre

Fig. S7. Meso-scale whole-brain mapping of neuronal inputs to MCH neurons traced using a low titer of ΔG rabies virus in late-symptomatic mice.

A-D. High-resolution quantification of monosynaptic inputs to MCH neurons across 130 brain areas of the hypothalamus (**A**), cerebral nuclei (**B**), cortex (**C**) and midbrain (**D**). Data are represented as the mean ± SEM of the proportion of inputs from each area/region in early-symptomatic *WT*;*Pmch*-Cre and *SOD1*^{G93A};*Pmch*-Cre mice (N=5 per genotype) injected with a low titer of modified rabies virus (LT). **e**. Schematic showing the relative contributions of four large-scale areas inputs to MCH neurons. The size of each circle represents the proportion of total whole-brain inputs and the arrows indicate unchanged (thin line), decreased (dashed line), or increased (thick line) large-scale inputs in *SOD1*^{G93A};*Pmch*-Cre mice. Anatomical abbreviations are based on the Allen Brain Atlas (ABA), and are fully defined in **Table S3**.

Figure S8

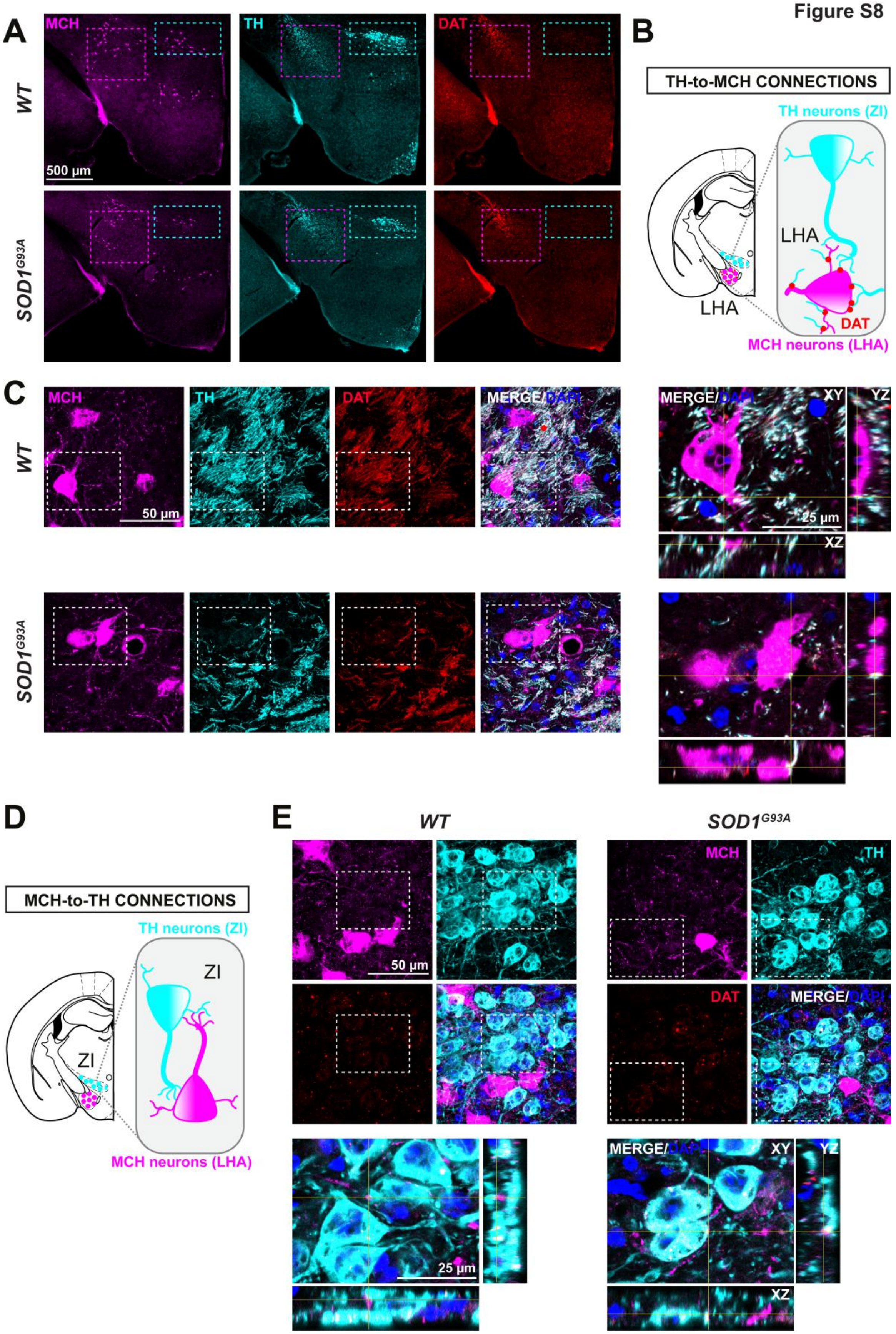
A
MCH
TH
DAT
WT
SOD1G93A
500 µm
B
TH-to-MCH CONNECTIONS
TH neurons (ZI)
LHA
LHA
DAT
MCH neurons (LHA)
C
MCH
TH
DAT
MERGE/DAPI
XY
YZ
XZ
50 µm
25 µm
WT
SOD1G93A
D
MCH-to-TH CONNECTIONS
TH neurons (ZI)
ZI
ZI
MCH neurons (LHA)
E
WT
SOD1G93A
MCH
TH
DAT
MERGE/DAPI
50 µm
25 µm
XY
YZ
XZ

Fig. S8. Bidirectional connections between TH and MCH neurons.

A. Representative images of the coronal section of the hypothalamus (overview) and of regions of interest: LHA (magenta dashed line square) and ZI (cyan dashed line square).

B. A schematic showing projections from TH (cyan) to MCH (magenta) neurons and the expression of the dopamine transporter - DAT (red) in the LHA.

C. Representative confocal images of the LHA showing MCH+ neurons surrounded by dense TH+/DAT+ fibres/puncta. The enlarged inset (white dashed line square) and the orthogonal XZ and YZ views illustrate the co-localization of the TH+/DAT+ fibres/puncta with the cell body of an MCH+ neuron.

D. Schematic depicting projections from the MCH (magenta) to TH (cyan) neurons in the ZI.

E. Representative confocal images of the ZI depicting TH+ neurons surrounded by MCH+ fibres. The enlarged inset (white dashed line square) and the orthogonal XZ and YZ views illustrate the co-localization of MCH+ fibres with the cell body of a TH+ neuron.

Scale bar: Overview images 1000 μm. Confocal images 50 μm (for lower magnification images) and 25 μm (for higher magnification images).

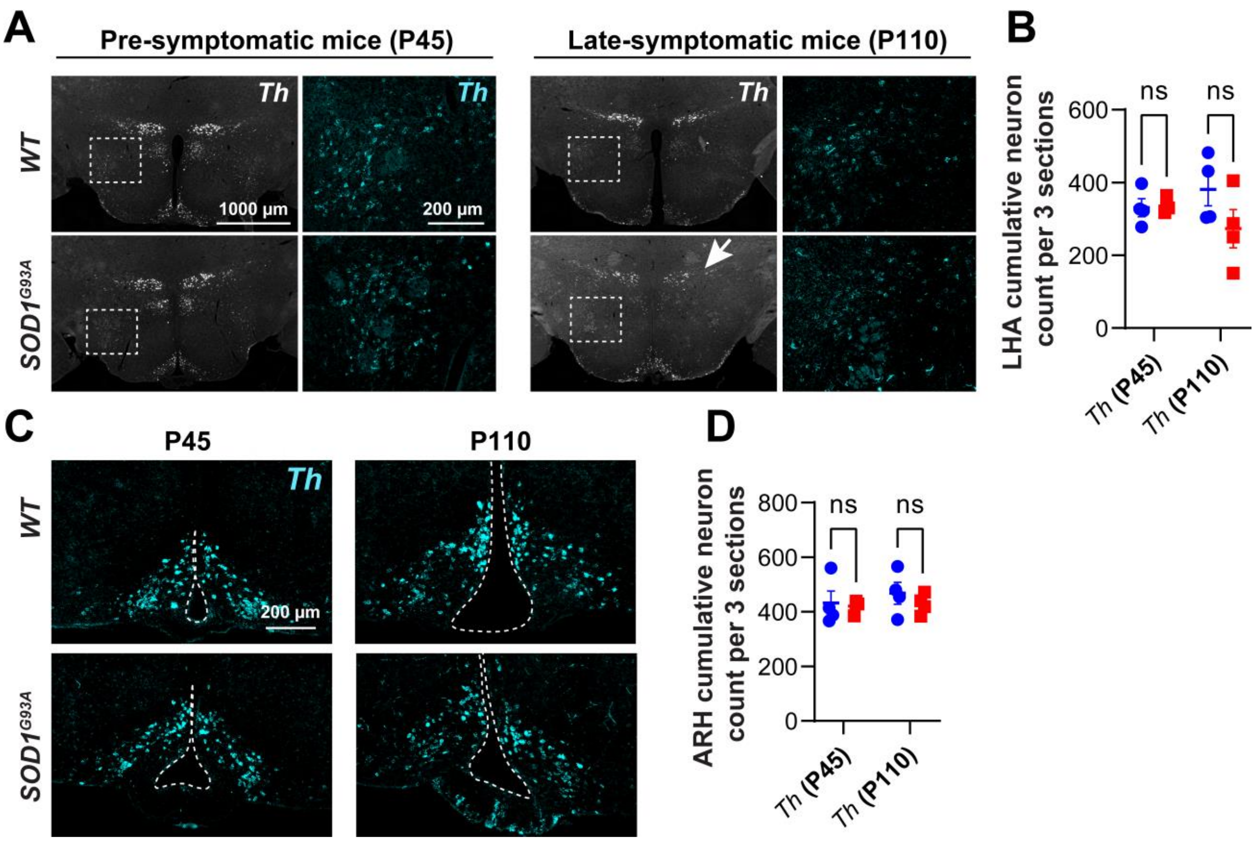

Fig. S9. DAergic neurons are preserved in the LHA and ARH.

A. Representative images show the distribution of *Th*+ neuronal populations (cyan) in the hypothalamus (overview) and in the LHA (white dashed line square) as detected by a RNAScope in *WT* (upper panels) and *SOD1*^{G93A} (lower panels) mice at P45 (left columns), and at P110 (right columns). The white arrow indicates the visible loss of *Th*+ neurons in the ZI of *SOD1*^{G93A} mice in the same section.

B. The graph shows the cumulative number of *Th*+ neurons in the LHA counted across three sections in *WT* (blue) and *SOD1*^{G93A} (red) mice.

C. Distribution of the *Th*+ neurons in the ARH around the third ventricle (white dashed line).

D. Quantification of *Th*+ neurons in the ARH.

N=4 animals per genotype. (ns) p-value>0.05. Multiple unpaired t-test per age. Data are presented as the mean ± SEM, with each data point representing a single animal. Scale bars: 1000 µm (for lower magnification images) and 200 µm (for higher magnification images).

Figure S10

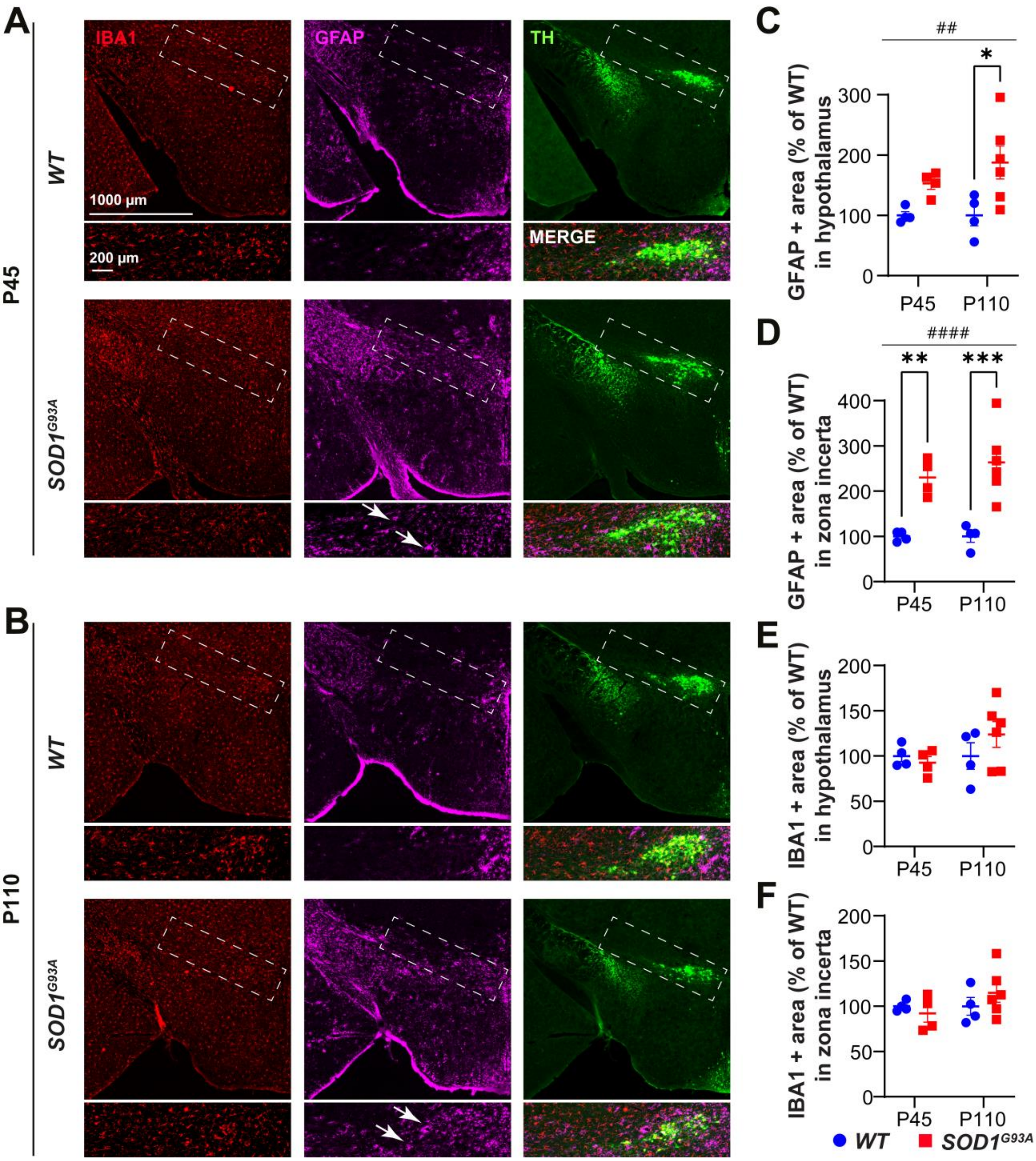

Fig. S10. Marked astrogliosis in the ZI occurs early, in pre-symptomatic *SOD1^{G93A}* mice.

A-B. Representative images of coronal hypothalamic sections (overview) and the ZI (enlarged insets – white dashed line square) showing immunolabelling for the microglia marker IBA1(red), astrocytes (GFAP, magenta) and DAergic TH+ neurons (green) in *WT* mice (upper panel) and high

GFAP expression in *SOD1*^{G93A} mice (lower panels) at P45 (**A**) and at P110 (**B**). The enlarged insets illustrate the increased density of GFAP+ cells in close proximity to TH+ neurons (white arrows).
C-D. Quantification plots show a significant increase in the percentage of GFAP+ area throughout the HY (**C**) in *SOD1*^{G93A} mice compared to *WT* littermates (p=0.02 at P110, and genotype effect by 2-way ANOVA ($F_{1,14}$=10.90, p=0.005)), which is particularly pronounced in the ZI (**D**) at both ages (p=0.006 at P45; p=0.0004 at P110, and genotype effect by 2-way ANOVA ($F_{1,14}$=35.77, p<0.0001)).
E-F. Graphs show a comparable percentage of IBA1+ area in the HY (**E**) and ZI (**F**) between *SOD1*^{G93A} and *WT* mice at both ages.
N=3-6 animals per genotype. 2-way ANOVA followed by Bonfferoni's multiple comparisons test, (##) p-value<0.01, (####) p-value<0.0001 for genotype effect and (*) p-value<0.05, (**) p-value<0.01, (***) p-value<0.001 for pairwise comparison. Data are presented as the mean ± SEM, with each data point representing a single animal. Scale bars: 1000 µm (for lower magnification images) and 200 µm (for higher magnification images)

Figure S11

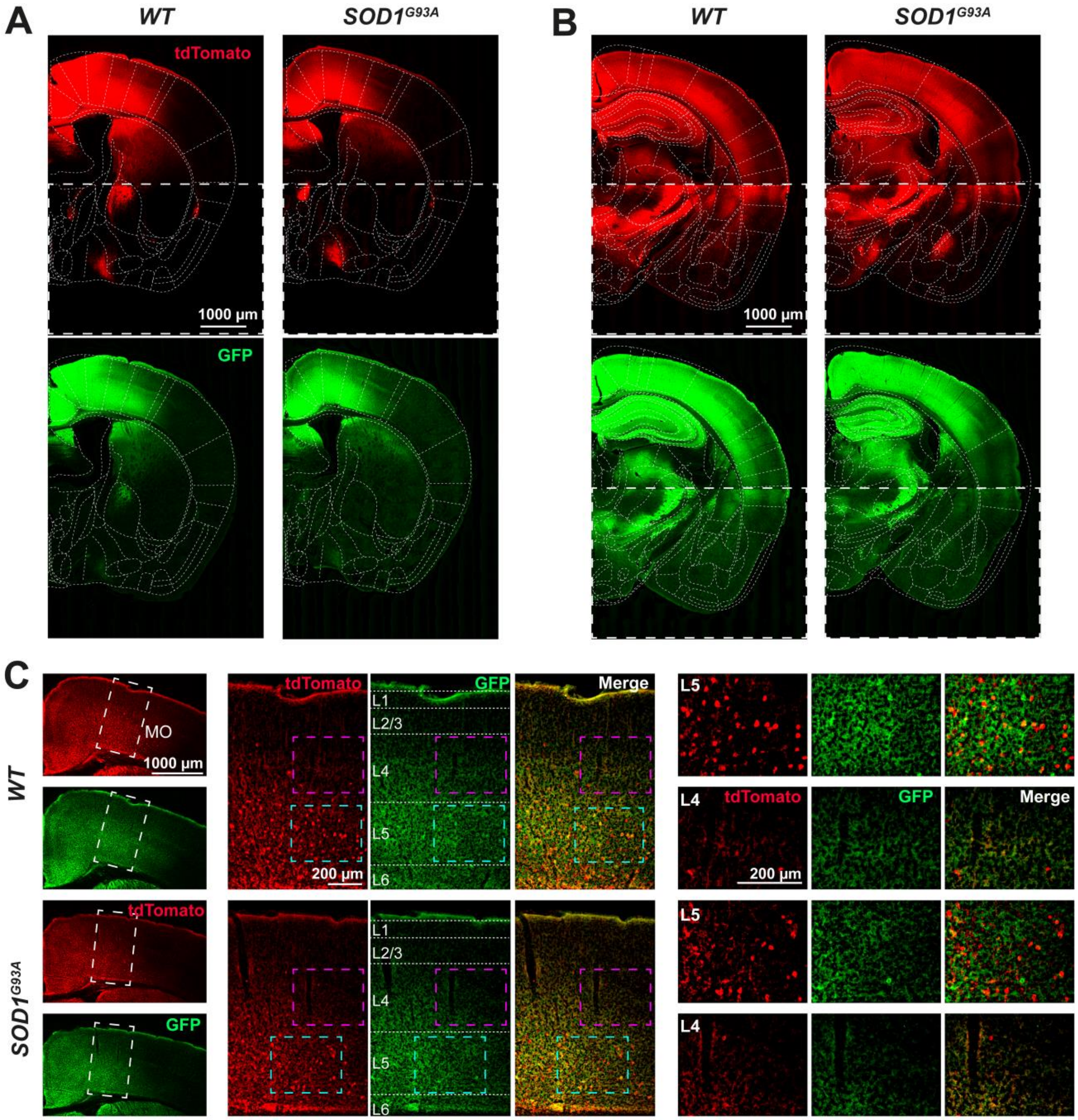

Fig. S11. Expression of anterograde viral tracer in the motor cortex.

A. Representative immunofluorescence (IF) images of coronal brain sections from *WT* and *SOD1*^{G93A} mice corresponding to the injection site (AP: -0.1 mm, ML: -0.7 mm, DV: -0.7 mm from Bregma), showing the expression of the fluorescent proteins tdTomato (red) and GFP (green), delivered by viral vectors, as indicated in Figure 8A. The white dashed line separates regions of the same section that were imaged using different exposure parameters.

B. Representative IF images of coronal brain sections used to investigate L5 PN connections to the hypothalamus corresponding to -2.0 mm and -1.6 mm caudal from Bregma.

C. Representative images of coronal sections of the mouse cortex (overview) and motor cortex (MO) (white dashed line square) showing tdTomato+ neurons and GFP+ puncta in layers 5 and 6 (cyan dashed line square), and the absence of these in layer 4 (magenta dashed line square) in both *WT* and $SOD1^{G93A}$ mice.

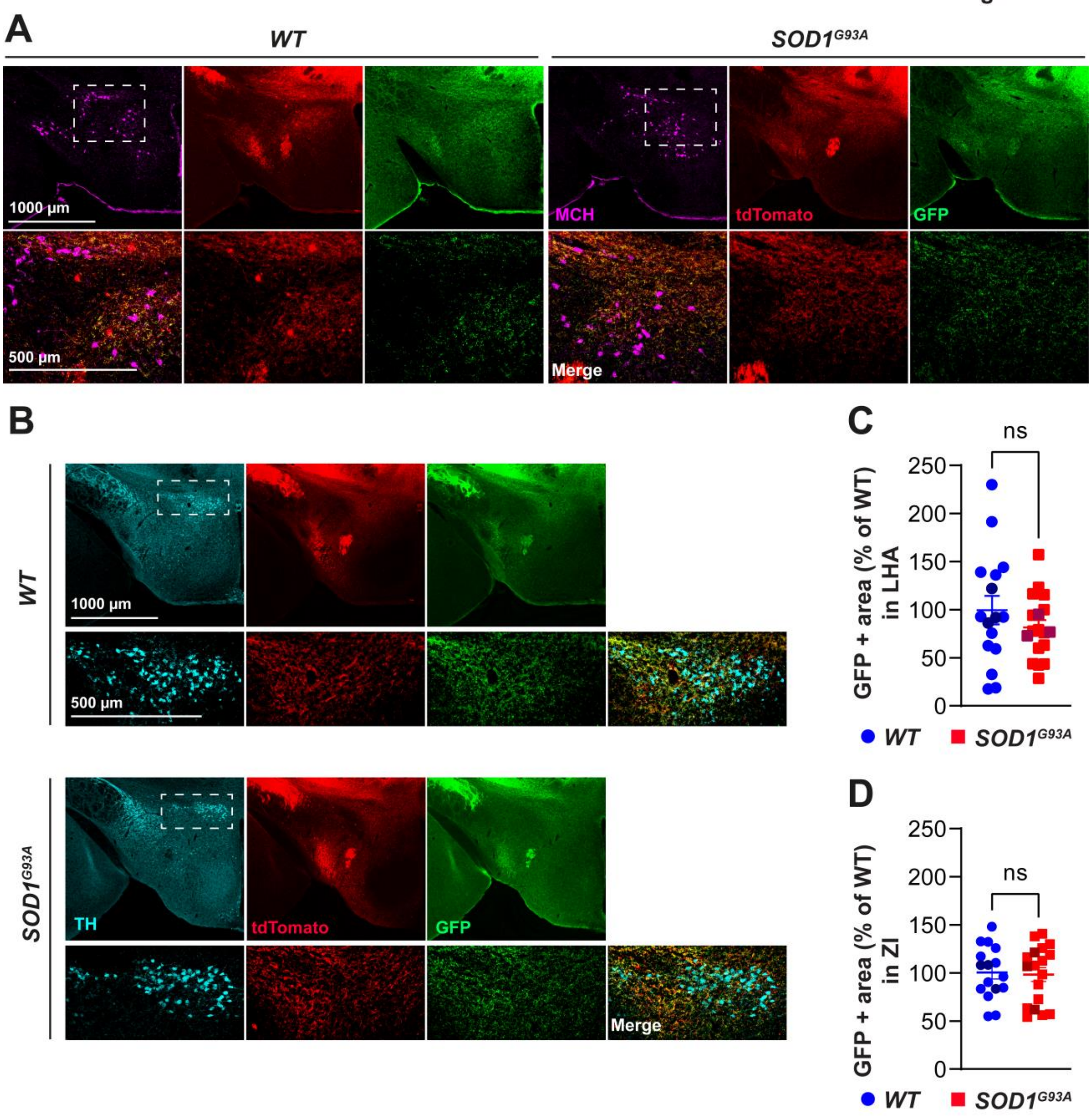

Fig. S12. Distribution of anterogradely traced cortical projections in the hypothalamus.

A-B. Representative IF images of coronal hypothalamic section (overview) and the LHA (**A**) or the ZI (**B**) (enlarged insets – white dashed line square) showing the distribution of tdTomato+ fibres (red) and GFP+ puncta (green) around MCH+ neurons (magenta) (**A**) and around TH+ neurons (cyan) (**B**) in *WT* and *SOD1*^{G93A} mice.

C-D. Quantification plots showing comparable percentage of the GFP+ area in the LHA (**C**) and in the ZI (**D**) between *SOD1*^{G93A} and *WT* mice.

N=3 animals per genotype. Two-tailed unpaired Student's t-test, (ns) p-value>0.05. Data are displayed as the mean ± SEM, with each data point representing a single animal. Scale bars: 1000 µm (for lower magnification images), 100 µm (for higher magnification images) and 50 µm for confocal images.

Table S1. Catalogue numbers of the RNA probes and dilutions used for the RNAscope assay

**Neuropetidergic population**	**Gene**	**Company**	**Channel**	**Catalog nr.**	**Dilution**
Melanin-concentrating hormone (MCH)	*Pmch*	ACD	2	478721-C2	1:100
Orexin/hypocretin	*Hcrt*	ACD	2	490461-C2	1:100
Cocaine- and amphetamine-regulated transcript (CART)	*Cartpt*	ACD	1	42001-C1	1:50
Tyrosine hydroxylase	*Th*	ACD	3	317621-C3	1:50 or 1:100 (Fig.8C; Suppl.Fig.)
Galanin	*Gal*	ACD	1	400961-C1	1:50
Agouti-related peptide	*Agrt*	ACD	2	400711-C2	1:50
Neuropeptide Y	*Npy*	ACD	1	313321-C1	1:50
Pro-opiomelanocortin (POMC)	*Pomc*	ACD	2	314081-C2	1:50
Somatostatin	*Sst*	ACD	1	404631-C1	1:50
Tdtomato	*Tdtomato*	ACD	2	317041-C2	1:100
Gamma-aminobutyric acid (GABA) vesicular transporter (VGAT)	*Slc32a1*	ACD	3	319191-C3	1:100
Vesicular glutamate transporter 2 (VGlut2)	*Slc17a6*	ACD	3	319171-C3	1:100

Table S2. Catalogue numbers of the antibodies and dilutions used for immunolabelling

**Primary antibodies**	**Host**	**Company**	**Catalog nr.**	**Dilution (application)**
MCH	Rabbit	Phoenix Pharma	H-070-47	1:200 (IF)
GFP	Chicken	Abcam	ab13970	1:500 (IF)
RFP	Goat	Origene	AB8181-200	1:500 (IF)
Tyrosine hydroxylase (TH)	Guinea pig	SySy	213 104	1:200 (IF)
DOPA-decarboxylase (DDC)	Mouse	SySy	369 011	1:200 (IF)
DOPA-decarboxylase (DDC)	Rabbit	SySy	369003	1:200 (IF)
B8H10-SOD1 (human)	Mouse	Medimabs	MM-0070	1:500 (IF)
Iba1	Rabbit	SySy	234008	1:1000 (IF)
GFAP	Mouse	Sigma	G3893	1:400 (IF)
Matrin3	Rabbit	Abcam	84422	1:250 (IF)
Dopamine transporter (DAT)	Guinea pig	SySy	284 005	1:200 (IF)
Tyrosine hydroxylase (TH)	Chicken	SySy	213 106	1:200 (IF)

**Secondary antibodies**	**Host**	**Company**	**Catalog nr.**	**Dilution (application)**
Anti-chicken CF 488A	Donkey	Sigma	SAB4600031	1:200 (IF)
Anti-goat AF 568	Donkey	Invitrogen	A11057	1:500 (IF)
Anti-Rabbit AF 647	Donkey	Invitrogen	A31573	1:500 (IF)
Anti-guinea pig AF 488	Donkey	Jackson	706-545-148	1:500 (IF)
Anti-mouse AF 568	Donkey	Invitrogen	A10037	1:500 (IF)
Anti-Rabbit AF 568	Donkey	Invitrogen	A10042	1:500 (IF)
Anti-mouse AF Plus 488	Donkey	Invitrogen	A32766	1:500 (IF)
Anti-guinea pig AF 647	Donkey	Jackson	706-605-148	1:500 (IF)
Anti-mouse AF 647	Donkey	Invitrogen	A31571	1:500 (IF)
Anti-guinea pig CF 568	Donkey	Sigma	SAB4600469	1:500 (IF)
Anti-chicken CF 488A	Donkey	Sigma	SAB4600031	1:500 (IF)

Table S3. Anatomical abbreviations of meso-scale whole-brain structures (acronym based on the Allen Atlas)

**Hypothalamus**	
	acronym
Dopaminergic A13 group	**A13**
Anterodorsal preoptic nucleus	**ADP**
Anterior hypothalamic nucleus	**AHN**
Arcuate hypothalamic nucleus	**ARH**
Anteroventral preoptic nucleus	**AVP**
Anteroventral periventricular nucleus	**AVPV**
Dorsomedial nucleus of the hypothalamus	**DMH**
Lateral mammillary nucleus	**LM**
Lateral preoptic area	**LPO**
Median preoptic nucleus	**MEPO**
Medial mammillary nucleus	**MM**
Medial preoptic nucleus	**MPN**
Medial preoptic area	**MPO**
Posterior hypothalamic nucleus	**PH**
Dorsal premammillary nucleus	**PMd**
Ventral premammillary nucleus	**PMv**
Parastrial nucleus	**PS**
Preparasubthalamic nucleus	**PST**
Parasubthalamic nucleus	**PSTN**
Pariventricular hypothalamic nucleus	**PV**
Paraventricular hypothalamic nucleus	**PVH**
Retrochiasmatic area	**RCH**
Subparaventricular zone	**SBPV**
Suprachiasmatic nucleus	**SCH**
Supraoptic nucleus	**SO**
Subthalamic nucleus	**STN**
Supramammillary nucleus, lateral	**SUMl**
Supramammillary nucleus, medial	**SUMm**
Tuberomammillary nucleus, dorsal part	**TMd**
Tuberomammillary nucleus, ventral part	**TMv**
Tuberal nucleus	**TU**
Ventrolateral preoptic nucleus	**VLPO**
Ventromedial hypothalamic nucleus	**VMH**
Zona incerta	**ZI**

**Cerebral nuclei**	
Anterior amygdalar are	**AAA**
Nucleus accumbens	**ACB**
Bed nucleus of the accessory olfactory tract	**BA**
Bed nuclei of the stria terminalis	**BST**
Central amygdalar nucleus	**CEA**
Caudoputamen	**CP**
Globus pallidus, external segment	**GPe**
Globus pallidus, internal segment	**GPi**
Lateral septal nucleus	**LS**
Magnocellular nucleus	**MA**
Medial amygdalar nucleus	**MEA**
Medial septal nucleus	**MS**
Diagonal band nucleus	**NDB**
Olfactory tubercle	**OT**
Striatum-like amygdalar nuclei	**sAMY**
Septofimbrial nucleus	**SF**
Substantia innominata	**SI**
Triangular nucleus of septum	**TRS**
**Cortex**	
Anterior cingulate area	**ACA**
Anterior cingulate area	**AI**
Auditory areas	**AUD**
Basolateral amygdalar nucleus	**BLA**
Basomedial amygdalar nucleus	**BMA**
Claustrum	**CLA**
Cortical amygdalar area	**COA**
Dorsal peduncular area	**DP**
Ectorhinal area	**ECT**
Entorhinal area	**ENT**
Endopiriform nucleus	**EP**
Gustatory areas	**GU**
Infralimbic area	**ILA**
Primary motor area	**MOp**
Secondary motor area	**MOs**
Nucleus of the lateral olfactory tract	**NLOT**
Orbital areaaud	**ORB**
Posterior amygdalar nucleus	**PA**
Piriform-amygdalar area	**PAA**
Perirhinal area	**PERI**

Piriform area	**PIR**
Prelimbic area	**PL**
Posterior parietal association areas	**PTLp**
Retrosplenial area	**RSP**
Primary somatosensory area	**SSp**
Supplemental somatosensory area	**SSs**
Subiculum, dorsal part	**SUBd**
Subiculum, ventral part	**SUBv**
Taenia tecta	**TT**
Visceral area	**VISC**
**Midbrain**	
Anterior pretectal nucleus	**APN**
Central linear nucleus raphe	**CLI**
Cuneiform nucleus	**CUN**
Dorsal nucleus raphe	**DR**
Edinger-Westphal nucleus	**EW**
Interpeduncular nucleus	**IPN**
Medial pretectal area	**MPT**
Midbrain reticular nucleus	**MRN**
Nucleus of the brachium of the inferior colliculus	**NB**
Nucleus of Darkschewitsch	**ND**
Nucleus of the posterior commissure	**NPC**
Periaqueductal gray	**PAG**
Pedunculopontine nucleus	**PPN**
Rostral linear nucleus raphe	**RL**
Red nucleus	**RN**
Midbrain reticular nucleus, retrorubral area	**RR**
Nucleus sagulum	**SAG**
Superior colliculus, motor related	**SCm**
Superior colliculus, sensory related	**SCs**
Substantia nigra, compact part	**SNc**
Substantia nigra, reticular part	**SNr**
Ventral tegmental area	**VTA**
Ventral tegmental nucleus	**VTN**
